# Spatial and Temporal Pattern of Ischemia and Abnormal Vascular Function Following Traumatic Brain Injury

**DOI:** 10.1001/jamaneurol.2019.3854

**Published:** 2019-11-11

**Authors:** Yoann Launey, Tim D. Fryer, Young T. Hong, Luzius A. Steiner, Jurgens Nortje, Tonny V. Veenith, Peter J. Hutchinson, Ari Ercole, Arun K. Gupta, Franklin I. Aigbirhio, John D. Pickard, Jonathan P. Coles, David K. Menon

**Affiliations:** 1 Division of Anaesthesia, Department of Medicine, Addenbrooke’s Hospital, University of Cambridge, Cambridge, United Kingdom; 2 Department of Anaesthesia and Critical Care Medicine, Centre Hospitalier Universitaire de Rennes, Rennes, France; 3 Wolfson Brain Imaging Centre, Department of Clinical Neurosciences, Addenbrooke’s Hospital, University of Cambridge, Cambridge, United Kingdom; 4 Department of Anaesthesiology, University Hospital Basel, Basel, Switzerland; 5 Department of Clinical Research, University of Basel, Basel, Switzerland; 6 Department of Anaesthesia, Norfolk and Norwich University Hospitals National Health Service Foundation Trust, Norwich, United Kingdom; 7 Birmingham Acute Care Research Group, Department of Critical Care Medicine, Queen Elizabeth Hospital, University of Birmingham, Birmingham, United Kingdom; 8 Division of Neurosurgery, Department of Clinical Neurosciences, Addenbrooke’s Hospital, University of Cambridge, Cambridge, United Kingdom

## Abstract

**Importance:**

Ischemia is an important pathophysiological mechanism after traumatic brain injury (TBI), but its incidence and spatiotemporal patterns are poorly characterized.

**Objective:**

To comprehensively characterize the spatiotemporal changes in cerebral physiology after TBI.

**Design, Setting, and Participants:**

This single-center cohort study uses ^15^oxygen positron emission tomography data obtained in a neurosciences critical care unit from February 1998 through July 2014 and analyzed from April 2018 through August 2019. Patients with TBI requiring intracranial pressure monitoring and control participants were recruited.

**Exposures:**

Cerebral blood flow (CBF), cerebral blood volume (CBV), cerebral oxygen metabolism (CMRO_2_), and oxygen extraction fraction.

**Main Outcomes and Measures:**

Ratios (CBF/CMRO_2_ and CBF/CBV) were calculated. Ischemic brain volume was compared with jugular venous saturation and brain tissue oximetry.

**Results:**

A total of 68 patients with TBI and 27 control participants were recruited. Results from 1 patient with TBI and 7 health volunteers were excluded. Sixty-eight patients with TBI (13 female [19%]; median [interquartile range (IQR)] age, 29 [22-47] years) underwent 90 studies at early (day 1 [n = 17]), intermediate (days 2-5 [n = 54]), and late points (days 6-10 [n = 19]) and were compared with 20 control participants (5 female [25%]; median [IQR] age, 43 [31-47] years). The global CBF and CMRO_2_ findings for patients with TBI were less than the ranges for control participants at all stages (median [IQR]: CBF, 26 [22-30] mL/100 mL/min vs 38 [29-49] mL/100 mL/min; *P* < .001; CMRO_2_, 62 [55-71] μmol/100 mL/min vs 131 [101-167] μmol/100 mL/min; *P* < .001). Early CBF reductions showed a trend of high oxygen extraction fraction (suggesting classical ischemia), but this was inconsistent at later phases. Ischemic brain volume was elevated even in the absence of intracranial hypertension and highest at less than 24 hours after TBI (median [IQR], 36 [10-82] mL), but many patients showed later increases (median [IQR] 6-10 days after TBI, 24 [4-42] mL; across all points: patients, 10 [5-39] mL vs control participants, 1 [0-3] mL; *P* < 001). Ischemic brain volume was a poor indicator of jugular venous saturation and brain tissue oximetry. Patients’ CBF/CMRO_2_ ratio was higher than controls (median [IQR], 0.42 [0.35-0.49] vs 0.3 [0.28-0.33]; *P* < .001) and their CBF/CBV ratio lower (median [IQR], 7.1 [6.4-7.9] vs 12.3 [11.0-14.0]; *P* < .001), suggesting abnormal flow-metabolism coupling and vascular reactivity. Patients’ CBV was higher than controls (median [IQR], 3.7 [3.4-4.1] mL/100 mL vs 3.0 [2.7-3.6] mL/100 mL; *P* < .001); although values were lower in patients with intracranial hypertension, these were still greater than controls (median [IQR], 3.7 [3.2-4.0] vs 3.0 [2.7-3.6] mL/100 mL; *P* = .002), despite more profound reductions in partial pressure of carbon dioxide (median [IQR], 4.3 [4.1-4.6] kPa vs 4.7 [4.3-4.9] kPa; *P* = .001).

**Conclusions and Relevance:**

Ischemia is common early, detectable up to 10 days after TBI, possible without intracranial hypertension, and inconsistently detected by jugular or brain tissue oximetry. There is substantial between-patient and within-patient pathophysiological heterogeneity; ischemia and hyperemia commonly coexist, possibly reflecting abnormalities in flow-metabolism coupling. Increased CBV may contribute to intracranial hypertension but can coexist with abnormal CBF/CBV ratios. These results emphasize the need to consider cerebrovascular pathophysiological complexity when managing patients with TBI.

## Key Points

**Question:**

How does ^15^oxygen positron emission tomography characterization of cerebral physiology after traumatic brain injury inform clinical practice?

**Findings:**

In this single-center observational cohort study of 68 patients and 27 control participants, early ischemia was common in patients, but hyperemia coexisted in different brain regions. Cerebral blood volume was consistently increased, despite low cerebral blood flow.

**Meaning:**

Per this analysis, pathophysiologic heterogeneity indicates that bedside physiological monitoring with devices that measure global (jugular venous saturation) or focal (tissue oximetry) brain oxygenation should be interpreted with caution.

## Teaser

This single-center observational cohort study aims to comprehensively characterize the spatiotemporal changes in cerebral physiology after traumatic brain injury by comparing a cohort of affected patients with healthy control participants.

## Introduction

Outcome after traumatic brain injury (TBI) is associated with not only initial injury severity but also secondary insults, including ischemia, brain swelling, and intracranial hypertension.^1^ Changes in cerebrovascular physiology after TBI contribute to all of these insults. Ensuring that cerebral blood flow (CBF) is adequate for oxygen and substrate delivery represents a fundamental management aim, which is pursued by optimizing cerebral perfusion pressure (CPP) and ventilation and controlling intracranial pressure (ICP).^2^

The physiology that underpins these aims and therapies used to achieve them are incompletely understood. Many studies have measured CBF without reference to regional cerebral oxygen metabolism (CMRO_2_), making it difficult to distinguish ischemia from CBF appropriately coupled to reduced metabolism and neuronal loss.^3,4,5^ Where studies have measured CMRO_2_,^6,7,8,9,10^ sample sizes have been small and do not cover the dynamic temporal and spatial evolution of cerebrovascular pathophysiology.^11,12,13,14,15,16^ Further, many studies have concentrated on physiology within lesions and have not examined regions that initially appear structurally normal.^17,18,19,20^

While brain swelling and ICP elevation drive up to half of TBI-associated mortality^21^ and remain therapeutic targets, the underlying physiology is unclear. Studies suggest that intracranial hypertension is predominantly attributable to brain edema, with cerebral blood volume (CBV) playing a limited role.^22^ However, in these studies, CBV was indirectly calculated rather than directly measured. These issues are important, since ICP-lowering therapies have competing benefits and harms. For example, lowering the partial pressure of carbon dioxide (Paco_2_) reduces vascular caliber and hence CBV and ICP, but it also reduces CBF,^23^ increasing the risk of ischemia.

While cerebrovascular physiology can be assessed with several imaging techniques,^5^ ^15^oxygen positron emission tomography (^15^O PET) is the current reference standard for CBF, CBV, CMRO_2_, and oxygen extraction fraction (OEF).^24^ While results from multiple centers^6,11,12,18^ have been integrated,^25^ this is hampered by differences in PET methodology (eg, bolus vs steady-state techniques)^24^ and approaches to data analysis (eg, voxel-based vs region-of-interest analysis).^6,11,12,18^ There remains a need for comprehensive characterization of cerebrovascular physiology after TBI using standardized ^15^O PET in a large patient sample across the full temporal disease narrative.

We aimed to comprehensively characterize cerebrovascular physiology within brain regions that initially appear structurally normal, using standardized ^15^O PET in a large patient cohort up to 10 days after TBI. We describe temporal changes in physiology, document changes in flow-metabolism coupling (CBF/CMRO_2_) and local microvascular flow-volume associations (CBF/CBV), disentangle ischemia from coupled hypoperfusion, and assess the contribution of vascular engorgement to ICP elevation.

## Methods

Additional details are provided within the eMethods in the Supplement. Control participants and patients with TBI requiring ICP monitoring within intensive care underwent ^15^O PET between February 1998 and July 2014. We excluded technically inadequate imaging studies and those with large variations of arterial pressure or Paco_2_. Some data have been reported previously,^4,6,14,19,23^ but none for the aims of the current article.

Studies were approved by the Cambridgeshire Research Ethics Committee and UK Administration of Radioactive Substances Advisory Committee and conducted in accordance with 1964 Declaration of Helsinki and later amendments. Volunteers provided informed consent, and assent was obtained from patient representatives, with patient consent obtained at follow-up if capacity was regained.

### Clinical Protocols

#### Patients

Protocol-driven therapy targeted an ICP of less than 20 mm Hg and a CPP of 60 to 70 mm Hg, as described by Menon.^26^ Mechanical ventilation and tight control of respiratory and cardiovascular physiology during PET studies with (where available) Paco_2_ reductions titrated against jugular venous saturation (SJVO_2_) and brain-tissue oximetry (BTPO_2_). Outcomes were recorded using the Glasgow Outcome Score (GOS)^27^ at 6 months after TBI.

### Imaging

Maps of CBF, CBV, CMRO_2_, and OEF were calculated as previously described^6,28,29^ after PET, using a General Electric Advance scanner (GE Medical Systems) at the Wolfson Brain Imaging Centre of the University of Cambridge (Cambridge, United Kingdom). We categorized studies into 3 groups: within 24 hours of TBI (early), 2 to 5 days after TBI (intermediate), and 6 to 10 days after TBI (late).

### Image Analysis

This analysis used whole-brain and standardized regions of interest (ROIs),^23^ excluding regions with lesions identified on registered computed tomography or magnetic resonance imaging. To account for pathophysiological heterogeneity within ROIs and assess ischemic burden, we estimated an individualized critical OEF threshold using a previously validated technique.^3,4,6^ Calculation of the volume of voxels with OEF greater than this threshold allowed estimation of the ischemic brain volume (IBV). We plotted IBV against SJVO_2_ and BTPO_2_ to identify thresholds for critical ischemia.

We calculated CBF/CMRO_2_ to assess the efficiency of flow-metabolism coupling and CBF/CBV as an index of local CPP.^30,31^ To determine whether differences in CBF/CBV represented intrinsic differences in vascular physiology or CPP variance, we calculated CBF/CBV divided by CPP.

### Statistical Analysis

Because data were not normally distributed, results are expressed as median (interquartile range [IQR]), and we used nonparametric statistical tests within R version 3.5.2 (R Foundation for Statistical Computing). In addition, we undertook linear mixed-effects regression of log-transformed PET parameters, adjusting for ROI, to analyze the difference in regional physiology between patients with TBI and healthy control participants, and we added time as a covariate (linear and quadratic to allow for nonlinear effects) to examine the association of time with changes in cerebrovascular physiology. We compared models with and without these terms using χ^2^ tests. Correlation coefficients were calculated using Spearman rank tests, and Mann-Whitney tests were used when comparing patients and control participants. For comparisons between groups, we used the Kruskal-Wallis test with the Dunn test for post hoc comparisons. Linear plots were used to help visualize the associations between parameters.

For between-group comparisons of basic demographic and clinical data, a *P* value of .05 or less was regarded as significant. Additional results were interpreted taking into consideration that, to infer statistical significance, a Bonferroni correction for 7 variables (CBF, CBV, CMRO_2_, OEF, CBF/CMRO_2_, CBF/CBV, and IBV) would require *P* < .007, and for regional comparisons (14 ROIs), *P* < 5.0 × 10^−4^ would be required. We also sought associations between IBV and GOS stratified for time after injury to replicate a previous finding^4^ in the early phase after TBI.

## Results

A total of 27 control participants and 68 patients with TBI were recruited; imaging studies from 7 control participants and 1 study from 1 patient with TBI were excluded because of poor quality. We analyzed data from 20 control participants with no clinically significant neurological or psychiatric illness and 90 PET sessions in 68 patients with TBI with stable physiology during imaging (eFigure 1 in the Supplement).

Included patients had severe TBI (Glasgow Coma Scale score ≤8 on admission) or presented with moderate TBI (Glasgow Coma Scale score, 9-12) and subsequently deteriorated. Of the 90 PET studies, 17 were within 24 hours of the TBI (early), 54 between 2 and 5 days after the TBI (intermediate), and 19 after 6 or more days after the TBI (late). Seventeen patients had PET studies on more than 1 occasion, but only 3 had studies at all 3 times. Table 1 summarizes the baseline demographics and physiology during imaging (median [IQR] age: control participants, 43 [31-47] years; patients with TBI, 29 [22-47] years; female participants: control participants, 5 of 20 [25%]; patients with TBI, 13 of 55 [19%]).

**Table 1.** Baseline Demographics and Physiology During Imaging

Characteristic	Finding, Median (IQR)		*P* Value^a^	Finding From Positron Emission Tomography Sessions in Patients With TBI, Median (IQR)			*P* Value^e^
	Control Participants (n = 20)	Patients With TBI (n = 68)		Early^b^ (n = 17)	Intermediate^c^ (n = 54)	Late^d^ (n = 19)	
Age, y	43 (31-47)	29 (22-47)	.05	36 (22-45)	28 (22-46)	29 (22-43)	.19
Sex							
Female	5	13	.57	4	14	1	.29
Male	15	55		13	40	18	
Postresuscitation Glasgow Coma Scale score	NA	7 (5-8)	NA	8 (5-10)	7 (5-7)	7 (5-9)	.55
Injury mechanism							
Car crash	NA	57	NA	10	33	14	NA
Fall	NA	26	NA	7	15	4	NA
Assault	NA	7	NA	0	6	1	NA
Marshall score^f^							
I	NA	0	NA	0	0	0	NA
II	NA	25	NA	1	17	8	NA
III	NA	5	NA	1	3	2	NA
IV	NA	0	NA	0	0	0	NA
V	NA	23	NA	8	21	6	NA
VI	NA	15	NA	7	13	3	NA
Primary lesion							NA
Extradural hematoma	NA	4	NA	2	2	2	NA
Subdural hematoma	NA	8	NA	4	8	1	NA
Contusion	NA	39	NA	11	32	11	NA
Diffuse axonal injury, intraventricular hemorrhage, and traumatic subarachnoid hemorrhage	NA	17	NA	0	12	5	NA
Injury severity score	NA	9 (7-17)	NA	8 (5-14)	9 (8-15)	12 (8-22)	.35
Acute Physiology and Chronic Health Evaluation II score	NA	9 (5-16)	NA	8 (5-11)	10 (5-13)	11 (9-21)	.09
Positron emission tomography time, h	NA	NA	NA	20 (15-21)	60 (43-84)	152 (136-182)	NA
Craniectomy	NA	23	NA	7	21	7	NA
Barbiturate coma	NA	7	NA	0	2	5	NA
Hemoglobin, g/dL	13.6 (13.1-14.6)	10.2 (9.5-11.1)	<.001	10.5 (9.7-11.0)^g^	10.1 (9.4-11.0)^g^	10.5 9.6-12.0)^g^	<.001
Partial pressure of oxygen, kPa^h^	11.4 (10.6-12.4)	15.2 (13.0-18.1)	<.001	15.5 (13.8-18.8)^g^	15.2 (13.1-18.0)^g^	14.3 (12.9-16.1)^g^	<.001
Partial pressure of carbon dioxide, kPa^h^	5.6 (5.2-5.7)	4.6 (4.2-4.8)	<.001	4.4 (3.9-4.8)^g^	4.7 (4.4-4.9)^g^	4.6 (4.5-4.8)^g^	<.001
Intracranial pressure, mm Hg^h^	NA	18 (13-22)		18 (14-20)	17 (13-22)	20 (15-22)	.69
Cerebral perfusion pressure, mm Hg^h^	81 (75-88)^i^	73 (70-80)	.008	73 (70-78)^j^	73 (69-78)^k^	80 (77-91)	.008
Glasgow Outcome Scale score	NA	4 (3-4)^l^	NA	NA	NA	NA	NA

Abbreviations: IQR, interquartile range; NA, not applicable; TBI, traumatic brain injury.

SI converstaion factor: To convert hemoglobin to g/L, multiply by 10.0.

^a^ Mann-Whitney test between all patients with TBI and control participants.

^b^ Within 24 hours of traumatic brain injury.

^c^ Two to 5 days after traumatic brain injury.

^d^ Six to 10 days after traumatic brain injury.

^e^ Kruskal-Wallis test between all groups, with subsequent comparisons made between each TBI group and control participants using the post hoc Dunn test.

^f^ Marshall Score: diffuse injury (I-IV), evacuated mass lesion (V), and nonevacuated mass lesion (VI).

^g^ *P* < .001; *P* values less than .05 were considered significant.

^h^ Intracranial pressure, cerebral perfusion pressure, partial pressure of oxygen, and partial pressure of carbon dioxide in individual imaging studies are the mean values of these variables over the course of each positron emission tomography study.

^i^ In control participants, cerebral perfusion pressure was defined assuming an intracranial pressure of 11 mm Hg.

^j^ *P* = .04; *P* values less than .05 were considered significant.

^k^ *P* = .09; *P* values less than .05 were considered significant.

^l^ In this group, 3 patients were missing data.

Patients had lower CPP (median [IQR], 73 [70-80] vs 81 [75-88]; *P* = .008) and Paco_2_ (median [IQR], 4.6 [4.2-4.8] kPa vs 5.6 [5.2-5.7] kPa; *P* < .001) than control participants. With the exception of CPP, which was significantly higher in the TBI group with late imaging compared with those with intermediate imaging (median [IQR] values, 80 [77-91] vs 73 [69-78] mm Hg; *P* = .008; Table 1), there were no differences in physiological variables (hemoglobin, Pao_2_, Paco_2_, and ICP) between the different TBI groups. Despite attempted management to a target ICP of 20 mm Hg or less, this was not achieved in all patients; 9 patients had ICP levels of 25 mm Hg or more during imaging.

### Global Physiology

Patients, compared with control participants, had lower CBF (median [IQR], 26 [22-30] mL/100 mL/min vs 38 [29-49] mL/100 mL/min; *P* < .001) and CMRO_2_ (median [IQR], 62 [55-71] μmol/100 mL/min vs 131 [101-167] μmol/100 mL/min; *P* < .001) and higher CBV (median [IQR], 3.7 [3.4-4.1] mL/100 mL vs 3.0 [2.7-3.6] mL/100 mL; *P* < .001) despite lower Paco_2_ (median [IQR], 4.6 [4.2-4.8] kPa vs 5.6 [5.2-5.7] kPa; *P* < .001). The CBF/CMRO_2_ ratio was higher in patients (median [IQR], 0.42 [0.35-0.49] vs 0.3 [0.28-0.33]; *P* < .001), while CBF/CBV was lower (median [IQR], 7.1 [6.4-7.9] vs 12.3 [11.0-14.0]; *P* < .001). The difference in OEF between patients and control participants (median [IQR], 39% [35%-43%] vs 44% [40%-45%]; *P* = .02) did not survive correction for multiple comparisons but was significantly lower at the intermediate point (median [IQR], 38% [32%-42%]; *P* = .004; Table 2). Compared with the rest of the group, physiology in patients who underwent craniectomy was similar to those who did not, while barbiturate coma was associated with lower CMRO_2_ (median [IQR], 53 [43-64] μmol/100 mL/min vs 65 [57-74] μmol/100 mL/min; *P* = .004), but reductions in CBF (median [IQR], 21 [18-27] mL/100 mL vs 27 [23-32] mL/100 mL; *P* = .01) and CBV (median [IQR], 3.2 [3.1-4.3] mL/100 mL vs 3.8 [3.4-4.1] mL/100 mL; *P* = .03) did not remain significant after correction for multiple comparisons.

**Table 2.** Temporal Pattern of Global Physiological Derangements

Positron Emission Tomography Sessions	Median (IQR)		*P* Value^a^	Patients With TBI, Median (IQR)			*P* Value
	Control Participants (n = 20)	TBI (n = 90)		Early (n = 17)^b^	Intermediate (n = 54)^c^	Late (n = 19)^d^	
Cerebral blood flow, mL/100 mL/min	38 (29-49)	26 (22-30)	<.001	25 (24-30)^e^	27 (23-32)^e^	24 (19-28)^e^	<.001
Cerebral blood volume, mL/100 mL	3.0 (2.7-3.6)	3.7 (3.4-4.1)	<.001	3.6 (3.3-3.9)	3.8 (3.5-4.1)^e^	3.5 (3.2-3.9)	<.001
Cerebral oxygen metabolism, μmol/100 mL/min	131 (101-167)	62 (55-71)	<.001	76 (70-85)^f^^,^^g^	61 (55-66)^e^	59 (47-66)^e^	<.001
Oxygen extraction fraction, %	44 (40-45)	39 (35-43)	.02	46 (41-52)^f^	38 (32-42)^e^	41 (35-43)	<.001
Cerebral blood flow–cerebral oxygen metabolism ratio	0.30 (0.28-0.33)	0.42 (0.35-0.49)	<.001	0.35 (0.28-0.39)^f^	0.44 (0.37-0.51)^e^	0.46 (0.33-0.48)^e^	<.001
Cerebral blood flow–cerebral blood volume ratio	12.3 (11.0-14.0)	7.1 (6.4-7.9)	<.001	7.2 (6.8-7.7)^e^	7.2 (6.4-8.0)^e^	6.5 (5.6-7.6)^e^	<.001
(Cerebral blood flow/cerebral blood volume)/cerebral perfusion pressure	0.18 (0.15-0.22)	0.09 (0.08-0.11)	<.001	0.09 (0.09-0.11)^e^	0.10 (0.09-0.12)^e^	0.08 (0.07-0.09)^e^	<.001
Ischemic brain volume, mL	1 (0-3)	10 (5-39)	<.001	36 (10-82)^e^	8 (4-16)^e^	24 (4-42)^e^	<.001
Patients with ischemic brain volume larger than the control range, %^h^	NA	39	NA	65	25	55	NA

Abbreviations: IQR, interquartile range; NA, not applicable; TBI, traumatic brain injury.

^a^ Mann-Whitney test between all patients with TBI and control participants.

^b^ Within 24 hours postinjury.

^c^ Days 2 through 5 postinjury.

^d^ Days 6 through 10 postinjury.

^e^ Kruskal-Wallis test between all groups, with the subsequent post hoc Dunn test surviving correction for multiple comparisons (*P* < .007) between each TBI group and control participants. Comparisons that did not meet this threshold are not shown.

^f^ Kruskal-Wallis test between all groups, with the subsequent post hoc Dunn test surviving correction for multiple comparisons (*P* < .007) between early and intermediate points after TBI. Comparisons that did not meet this threshold are not shown.

^g^ Kruskal-Wallis test between all groups, with the subsequent post hoc Dunn test surviving correction for multiple comparisons (*P* < .007) between early and late points after TBI. Comparisons that did not meet this threshold are not shown.

^h^ The percentage of patients with ischemic brain volume larger than the maximum value found in control participants (20 mL) are shown.

### Regional Physiology

**Figure 1.**
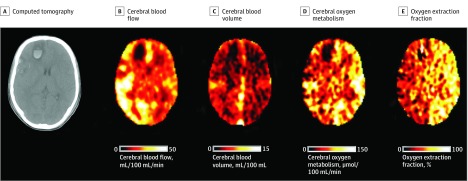
Spatial Variation in Regional Physiology After Traumatic Brain Injury Example findings from a patient with a traumatic brain injury on day 3 after injury. The patient’s initial Glasgow Coma Scale score was 12 but deteriorated on admission, requiring sedation and ventilation for management of intracranial pressure. Within the vicinity of the right frontal and temporal hemorrhagic contusions, cerebral blood flow and cerebral oxygen metabolism are decreased, but within the contralateral hemisphere, which appears structurally normal, cerebral blood flow is variably reduced and the oxygen extraction fraction is markedly increased, suggesting ischemia.

**Figure 2.**
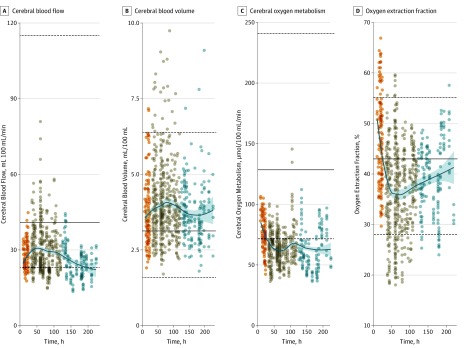
Spatial and Temporal Pattern of Regional Physiological Derangements in Patients With Traumatic Brain Injury Individual regional values for cerebral blood flow, blood volume, oxygen metabolism, and oxygen extraction fraction in patients with traumatic brain injury, plotted against time postinjury (orange, within 24 hours [early]; brown, days 2-5 [intermediate]; blue, days 6-10 [late]). The fitted blue lines represent modeling of the association between each parameter and hours postinjury using locally weighted scatterplot smoothing, with the 95% CIs shown in light blue. The solid and dashed black lines represent the median and the full range of values for healthy volunteers, respectively.

Spatial heterogeneity was obvious on imaging (Figure 1), with regional variability shown across the whole-brain ROI template (eFigure 2 in the Supplement) both within and between patients, compared with control participants (eTables 1 and 2 and eFigure 3 in the Supplement). Using a linear mixed-effects regression model comparing affected patients with control participants, regional CBF, CBV, and CMRO_2_ in patients were found to be significantly different (covariate effect estimates, −0.37, 0.16, and −0.71, respectively; all comparisons *P* < .001), while OEF (covariate effect estimate, −0.12; *P* = .01) did not remain significant after correction for multiple comparisons. There were also significant differences between control participants and patients at various points after injury (eTable 3 in the Supplement), and the time-dependent models for CBF, CBV, CMRO_2_, and OEF fit the data significantly better (−0.85, −0.39, 0.72, and 0.9 for standardized time, and 0.17, 0.09, −0.23, and 0.25 for standardized time^2^, respectively; all comparisons,* P* < .001). Temporal trends differed between patients and control participants (Figure 2): CBF was low, showed a transient recovery, and then decreased again, while CMRO_2_ was initially low and decreased further over time. Oxygen extraction fraction was initially high and fell in the intermediate period before trending toward normal values. Cerebral blood volume showed less variation and Cerebral blood volume showed less variation and showed a trend for higher values than normal at all points. The 17 patients who underwent PET on more than 1 occasion showed a pattern similar to the overall cohort (eTable 4 and eFigure 4 in the Supplement).

### Flow Metabolism Coupling

Wide variations in OEF (which imply large variations in flow-metabolism coupling) and regions with particularly low OEF (implying hyperemia) were common and most prominent between days 2 through 5 (Figure 2 and eFigure 3 within the Supplement). These abnormalities in CBF/CMRO_2_ ratios were quantified across ROIs in individual patients using the Spearman rank test, with high ρ values providing evidence of preserved flow-metabolism coupling. While there was substantial interparticipant heterogeneity, patients with TBI (median ρ, 0.44 [IQR, 0.25-0.64]) showed lower correlation coefficients than control participants (ρ, 0.75 [0.67-0.86]; *P* < .001; eFigure 5 and eTable 5 in the Supplement).

### Ischemia

**Figure 3.**
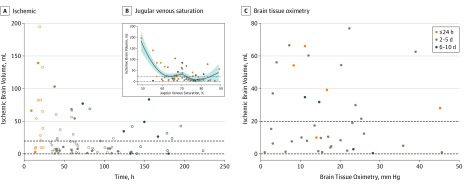
Burden of Cerebral Ischemia A, The ischemic brain volume in patients with traumatic brain injury is plotted against time postinjury (orange, within 24 hours [early]; brown, days 2-5 [intermediate]; blue, days 6-10 [late]). The dashed lines represent minimal and maximal values for ischemic brain volume in control participants. Open circles represent patients with intracranial pressure of 20 mm Hg or less, and filled circles represent patients with intracranial pressure greater than 20 mmHg. B, Association between the ischemic brain volume and jugular venous saturation in 63 patients who had jugular bulb catheters in place during ^15^oxygen positron emission tomography studies. Data are shown for studies within 24 hours (orange circles), days 2 through 5 (gray), and days 6 through 10 (blue) postinjury, with the blue line denoting the fit using a locally weighted scatterplot smoothing regression model, with the 95% CI shown in lighter blue. The dashed lines represent minimal and maximal values for ischemic brain volume in control participants. C, Association between ischemic brain volume and brain tissue oximetry. The ischemic brain volume in patients with traumatic brain injury is plotted against brain tissue oximetry in 38 patients with monitoring in place during ^15^oxygen positron emission tomography studies. Data are shown for studies within 24 hours (orange circles), days 2 through 5 (gray), and days 6 through 10 (blue) postinjury. The dashed lines represent minimal and maximal values for ischemic brain volume in control participants.

Oxygen extraction fraction showed a nonlinear association with CBF, with substantial within-participant and between-participant heterogeneity (eFigure 6 in the Supplement). Within 24 hours, OEF increased sharply when CBF was less than 25 mL/100 mL/min (the lower CBF limit in control participants), suggesting cerebral ischemia. Across all points, while some regions showed increases, OEF tended to plateau at approximately 45% when CBF was less than 25 mL/100 mL/min. While IBV elevations were most common within 24 hours of injury (median [IQR] IBV in early imaging, 36 [10-82] mL), high IBV was observed up to 10 days after TBI (median [IQR] in late imaging, 24 [4-42] mL) and were not associated with intracranial hypertension (Table 2; Figure 3). All patients had CPP greater than 60 mm Hg. Ischemic brain volume was not associated with CPP (ρ, –0.06; *P* = .60) and did not differ in those who underwent craniectomy (median [IQR], 11 [5-28] vs 10 [4-49] mL; *P* = .75) or barbiturate coma (median [IQR], 24 [5-67] vs 10 [4-37] mL; *P* = .36). Within 24 hours, IBV was associated with worse outcomes, as quantified via the GOS (ρ, –0.63; *P* = .006), but there was no association across the whole cohort (ρ, −0.06; *P* = .71). After TBI, there was no association between CBF and Paco_2_ (ρ, –0.39; *P* = .09) or CBF and IBV measured during PET (ρ, –0.17; *P* = .10) or within separate temporal cohorts.

### Bedside Monitors of Brain Oximetry

We had 67 PET sessions with SJVO_2_ and 38 with BTPO_2_ monitoring. We found no association between IBV and SJVO_2_ (Figure 3). Within the first 24 hours, there was some evidence of an inflection point at an SJVO_2_ of approximately60%; when the level was less than this, IBV showed progressive increases. However, there were patients with high IBV who showed SJVO_2_ values greater than the 60% threshold.

There was no association between IBV and BTPO_2_ (Figure 3). In fact, many individuals with BTPO_2_ values less than 15 mm Hg had IBV values within the control range.

### Cerebral Blood Flow and Volume

When compared with control participants, patients with TBI showed lower whole-brain CBF/CBV for all points (Table 2); in nonlesion ROIs, this was particularly true within intermediate and late points (eTable 2 in the Supplement). Correcting for lower CPP in patients compared with control participants (73 mg vs 81 mm Hg; *P* = .008 ) did not remove this difference. Though patients showed more variability in the CBF/CBV correlation, we observed no group level differences in ρ values between control participants and any TBI temporal subgroups (eFigure 7 and eTable 5 in the Supplement).

### Cerebral Blood Volume and Intracranial Pressure

Increases in ICP were found up to 10 days after TBI (Table 1) with CBV inversely associated with ICP (ρ, −0.23; *P* = .04; eFigure 8 in the Supplement), but no association between CBV and ICP after accounting for Paco_2_ during PET was found (ρ, −0.17; *P* = .13). There was a negative association between Paco_2_ and ICP (ρ, −0.39; *P* < .001), with lower Paco_2_ in patients with intracranial hypertension (median [IQR], 4.3 [4.1-4.6] kPa vs 4.7 [4.3-4.9] kPa; *P* = .001; eFigure 9 in the Supplement), presumably driven by hyperventilation therapy. Despite lower Paco_2_, CBV remained higher in patients with intracranial hypertension than control participants (median [IQR], 3.7 [3.2-4.0] vs 3.0 [2.7-3.6] mL/100 mL; *P* = .002; eFigure 9 in the Supplement). There was no association between CBV and Paco_2_ in all patients (ρ, 0.19; *P* = .07) or in those with ICP greater than 20 mm Hg (ρ, 0.03; *P* = .89).

## Discussion

To our knowledge, this is the largest ^15^O PET study in patients with TBI, and it shows clear phasic changes in cerebral physiology. Our results have clinical relevance in 3 areas. First, they map the presence, temporal course, and association of ischemia with outcome. Second, they elucidate the association between CBV increases and intracranial hypertension and the relevance of this association for ICP management. Finally, these data document abnormal flow-metabolism coupling and cerebral vasoregulation after TBI, which is a potential target for the development of future therapeutic interventions.

### Ischemic Burden

After TBI, CBF shows a triphasic pattern, classically described as consisting of early hypoperfusion (<24 hours), hyperemia (1-3 days), and vasospasm (after 3 days).^32^ Most previous analyses have been based on indirect CBF measurement, typically using transcranial Doppler ultrasonography. Imaging has not commonly used CMRO_2_ measurement,^5^ making it impossible to differentiate ischemia from hypoperfusion appropriately coupled with hypometabolism. Our data show that classical ischemia (diagnosed by the incontrovertible metabolic signature of high OEF) is seen in many patients within 24 hours of injury, can be observed later, and can occur despite optimization of CPP, Paco_2_, and ICP.

Other groups have found less consistent evidence of classical ischemia using ^15^O PET in patients with TBI.^12,17,18,25^ Several of these studies focused on patients with less severe TBI at later points, with less significant physiological derangements, and for a limited period. Some specifically addressed contused brain and provided little data on cerebrovascular pathophysiology in structurally normal brain, which often constitutes the largest tissue compartment after TBI. Additional contributors to this discrepancy include the use of bolus, as opposed to our use of steady-state PET techniques,^24^ and different approaches to quantifying ischemic burden (ROI vs voxel-based methods). Our data are in keeping with the frequent detection of cerebral ischemia and infarction after TBI, both with antemortem imaging^33^ and postmortem neuropathology.^34,35,36^

Hyperventilation can result in acute CBF reductions,^23^ with increases in OEF and reductions in CMRO_2_ in some patients.^23^ However, IBV in this cohort was not associated with steady-state Paco_2_, perhaps because hyperventilation was being titrated to monitors of cerebral oximetry (SJVO_2_ or BTPO_2_) and used to control intracranial hypertension.^37^ Alternatively, hyperventilation-induced ischemia may resolve over time as extracellular pH in the brain normalizes.^38^ Although this compensation is incomplete for CBF reductions (as opposed to CBV^39^), it may make it difficult to detect ischemia at steady-state low Paco_2_ levels (which was the case in the participants in this study).

### CBF-OEF Associations

Reductions in CBF to less than the normal range resulted in sharp OEF increases within the first 24 hours. However, such OEF increases were not systematically achieved by similar CBF reductions at later points, which could imply a failure of oxygen extraction or use. Microvascular dysfunction and diffusion hypoxia are known to occur in a normal-appearing brain of a patient with TBI^6,40^ and can limit the ability to increase OEF. Similarly, oxygen use may be prevented by mitochondrial dysfunction, either because of structural mitochondrial damage^7,41^ or competitive inhibition of the mitochondrial respiratory chain by nitric oxide.^42^ Finally, persistent CBF reductions may result in patchy necrosis or selective neuronal loss. The overall CBF in such heterogeneous regions may be low but appropriate for the remaining viable tissue.

### Abnormal Flow Metabolism Coupling and Bedside Monitoring of Ischemia

These data show generalized abnormalities in flow-metabolism coupling, with weaker associations between CBF and CMRO_2_ in patients with TBI compared with control participants. A high CBF/CMRO_2_ ratio implies hyperemia and was most prominent 2 to 5 days postinjury. These findings are consistent with transcranial Doppler ultrasonography data,^32^ but our additional CMRO_2_ measurement allows confirmation that these represent true hyperemia. We documented between-participant heterogeneity, but also within-participant heterogeneity, since regions with high and low OEF often coexisted within the same patient. This heterogeneity may confound bedside monitoring, since global methods (SJVO_2_) may dilute and miss focal pathophysiology, while focal monitors (BTPO_2_) are critically dependent on sensor position. We saw some evidence of an SJVO_2_ threshold of approximately 60%, and at levels less than this, IBV increased (particularly within 24 hours of TBI). However, many patients with high IBV had SJVO_2_ values that were substantially greater than this threshold level, and use of the typical SJVO_2 _less than 50%^43^ would have missed all but 1 patient with critical brain ischemia. We found no reliable association between BTPO_2_ and IBV. In fact, many participants with low BTPO_2_ values (<15 mm Hg) did not show IBV increases. Our inference is that focal BTPO_2_ monitoring is not reliably associated with the global or regional burden of ischemia.

### Cerebral Ischemia and Outcome

As demonstrated previously,^4^ IBV within the first 24 hours after TBI was associated with GOS, underlining the clinical significance of early ischemia. The lack of correlation between later IBV and GOS raises the possibility that later ischemia is better tolerated, but it is more likely that other energy failure mechanisms (such as diffusion hypoxia^6^ and mitochondrial dysfunction^7^) make greater contributions to outcome at these points.

### Association of CBV With ICP

Previous reports, using estimates based on a combination of xenon and perfusion CT,^21,22^ reported reduced CBV in patients with TBI. The direct CBV measurement in this report shows that it was consistently raised, but the contribution to ICP elevation was complex. Patients with intracranial hypertension showed lower CBV values than those with ICP values of 20 mm Hg or less, but this unexpected association was likely attributable to lower Paco_2_ in patients with intracranial hypertension, in whom hyperventilation therapy was used. Despite this, their CBV values remained significantly greater than those of control participants, suggesting that CBV elevations continued to contribute to intracranial hypertension. While cerebral edema is an important driver of intracranial hypertension, we show that CBV increases also contribute, providing a physiological basis for interventions aimed at reducing the vascular contribution to intracranial volume.

### CBF/CBV Associations

We found regional differences in CBF/CBV (eFigure 3 in the Supplement) in patients with TBI, replicating past results from control participants.^31^ Since experimental studies suggest a linear association between CPP and CBF/CBV,^30^ we calculated CBF/CBV divided by CPP to account for lower CPP values in individuals with TBI. However, CBF/CBV divided by CPP remained lower in individuals with TBI (Table 2), suggesting that simple autoregulatory vasodilatation could not explain this finding. Reduced CBF/CBV in individuals with TBI may represent impaired dilatation of precapillary resistance units (resulting in low CBF) and/or a disproportionate increase in (probably venous) capacitance in the cerebrovascular circuit. These inferences are concordant with past TBI data, which show that the effect of hyperventilation on CBV (and hence ICP) is transient, while the CBF reductions it produces are dominant and sustained.^39^

### Limitations

Although our data were collected prospectively in accordance with a common protocol, this was a retrospective collation with nonconsecutive recruitment driven by convenience and logistics, which makes generalizability difficult to assess. Imaging was not possible on days on which patients were too unstable or scanners were unavailable. Although data were acquired over 16 years, all patients were recruited after the introduction of the Cambridge ICP/CPP protocol, and any changes in physiological targets over the recruitment period were relatively small and have been detailed in a recent publication.^44^ Systemic physiology during each scan is available and explicitly summarized in Table 1. We used global, regional, and voxel-based analyses, but each has potential pitfalls; global and region-based measures may miss voxel-level pathophysiological heterogeneity, whereas voxel-based approaches are more susceptible to noise. While this was, to our knowledge, the largest single-center ^15^O PET study in individuals with TBI (90 scans), ideally an assessment of the temporal patterns of physiology would make use of sequential studies within participants. However, such studies are difficult to undertake and limited by considerations of patient safety and stability. Consequently, the sample available for analysis represents the data that were obtainable in this context. We did have 17 patients in whom PET were available on more than 1 occasion. The temporal patterns in this subset broadly replicate those of the overall cohort, suggesting the larger data set provides a useful representation of temporal trends in regional physiology. Further analysis, using a mixed-effects model that accounted for the inclusion of data from multiple ROIs within each participant, confirmed the association of time postinjury with regional cerebrovascular variables in comparison with data from control participants. Finally, this analysis explored pathophysiology in structurally normal tissue to examine the brain at risk of evolving injury, and hence it does not provide a complete picture of all pathophysiological tissue compartments after TBI.

## Conclusions

In a large cohort of individuals with TBI, ^15^O PET imaging shows systematic changes in cerebrovascular physiology that have direct clinical relevance. Early ischemia (<24 hours) occurs in approximately two-thirds of patients, is detectable up to 10 days postinjury, and is not limited to patients with intracranial hypertension. We found significant outcome associations with ischemia that occurred early but not after 24 hours, implying that other pathophysiological mechanisms of energy failure may be dominant during later phases of the disease narrative. We demonstrate substantial pathophysiological heterogeneity within patients, with ischemia and hyperemia coexisting in different brain regions, reflecting abnormalities in flow-metabolism coupling. Global (SJVO_2_) or focal (BTPO_2_) measures of cerebral oximetry provide data that guide clinical management, but physiological heterogeneity dictates that these should be interpreted with caution. Cerebral blood volume is consistently increased in patients with TBI and remains higher than control values even in patients with intracranial hypertension, despite lower Paco_2_ being used to manage ICP. These data are in keeping with CBV increases as a contributor to intracranial hypertension. However, there may be disassociation of CBF/CBV homeostasis, with disproportionately high CBV seen across the entire postinjury period despite low CBF. Such physiological dissociation, along with the regional variations in physiology and the inconsistent associations with monitoring that we demonstrate in this study, may inform clinical management of patients with TBI.

## Notes

Supplement. **eMethods.** **eReferences.** **eFigure 1.** Flowchart of oxygen-15 positron emission tomography data **eFigure 2.** Region of interest map **eFigure 3.** Regional variation in physiology after traumatic brain injury **eFigure 4.** Spatial and temporal pattern of regional physiological derangements in serial data obtained in patients after traumatic brain injury **eFigure 5.** Flow metabolism coupling **eFigure 6.** Scatterplots of association between oxygen extraction fraction and cerebral blood flow in patients after traumatic brain injury **eFigure 7.** Association between cerebral blood flow and blood volume **eFigure 8.** Intracranial hypertension and cerebral blood volume **eFigure 9.** Intracranial hypertension and hyperventilation **eTable 1.** Regional physiological derangements **eTable 2.** Temporal pattern of regional physiological derangements **eTable 3.** Linear mixed effects model for comparison of regional physiological derangements **eTable 4.** Temporal pattern of regional physiological derangements from serial data within patients **eTable 5.** Flow metabolism coupling and the association between cerebral blood flow and volume
