# Supplementary material for: Spatial and Temporal Pattern of Ischemia and Abnormal Vascular Function Following Traumatic Brain Injury
Source: JAMA Neurol. 2019 Nov 11;77(3):339–49. doi: 10.1001/jamaneurol.2019.3854 (PMC6865302; doi:10.1001/jamaneurol.2019.3854)
Supplement: Supplement. — eMethods. eReferences. eFigure 1. Flowchart of oxygen-15 positron emission tomography data eFigure 2. Region of interest map eFigure 3. Regional variation in physiology after traumatic brain injury eFigure 4. Spatial and temporal pattern of regional physiological derangements in serial data obtained in patients after traumatic brain injury eFigure 5. Flow metabolism coupling eFigure 6. Scatterplots of association between oxygen extraction fraction and cerebral blood flow in patients after traumatic brain injury eFigure 7. Association between cerebral blood flow and blood volume eFigure 8. Intracranial hypertension and cerebral blood volume eFigure 9. Intracranial hypertension and hyperventilation eTable 1. Regional physiological derangements eTable 2. Temporal pattern of regional physiological derangements eTable 3. Linear mixed effects model for comparison of regional physiological derangements eTable 4. Temporal pattern of regional physiological derangements from serial data within patients eTable 5. Flow metabolism coupling and the association between cerebral blood flow and volume [file jamaneurol-77-339-s001.pdf]

Supplementary Online Content

Launey Y, Fryer TD, Hong YT, et al. Spatial and temporal pattern of ischemia and abnormal vascular function following traumatic brain injury. *JAMA Neurol*. Published online November 11, 2019. doi:10.1001/jamaneurol.2019.3854

eMethods.

eReferences.

eFigure 1. Flowchart of oxygen-15 positron emission tomography data

eFigure 2. Region of interest map

eFigure 3. Regional variation in physiology after traumatic brain injury

eFigure 4. Spatial and temporal pattern of regional physiological derangements in serial data obtained in patients after traumatic brain injury

eFigure 5. Flow metabolism coupling

eFigure 6. Scatterplots of association between oxygen extraction fraction and cerebral blood flow in patients after traumatic brain injury

eFigure 7. Association between cerebral blood flow and blood volume

eFigure 8. Intracranial hypertension and cerebral blood volume

eFigure 9. Intracranial hypertension and hyperventilation

eTable 1. Regional physiological derangements

eTable 2. Temporal pattern of regional physiological derangements

eTable 3. Linear mixed effects model for comparison of regional physiological derangements

eTable 4. Temporal pattern of regional physiological derangements from serial data within patients

eTable 5. Flow metabolism coupling and the association between cerebral blood flow and volume

This supplementary material has been provided by the authors to give readers additional information about their work.

© 2020 Yoann Launey et al. *JAMA Neurology.*

eMethods

Clinical protocols

Protocolised and graded interventions used for cerebral perfusion pressure (CPP) and intracranial pressure (ICP) control included sedation (propofol 3-5 $mg \cdot kg^{-1} \cdot hr^{-1}$ and fentanyl 1-4$\mu g \cdot kg^{-1} \cdot hr^{-1}$) and neuromuscular blockade, surgery for space-occupying lesions, drainage of cerebrospinal fluid, vasoactive agents for CPP increment, osmotic therapy, mild hyperventilation (aimed at ~35 mmHg [4·5 kPa]), mild to moderate hypothermia (33–36°C), decompressive craniectomy, and barbiturate metabolic depression.

Healthy control participants

Blood pressure, pulse oximetry, and arterial blood gases were monitored to ensure physiologic stability during imaging. For calculating CPP in healthy control participants (HC), we assumed an ICP of 11mm Hg when lying supine.[1]

Imaging

Positron emission tomography (PET) data were acquired on a GE Advance PET scanner (GE Medical Systems, Waukesha, USA). Data were acquired in 3D mode for the last 10 minutes of a 20-minute steady state infusion of 800 MBq of ^{15}O water; in 3D mode for 5 minutes after a 1 minute inhalational of 750MBq of ^{15}O carbon monoxide; and in 2D mode for the last 10 minutes of a 20-minute steady state inhalation of 7,200 MBq of ^{15}O oxygen. Emission data were corrected for photon attenuation using data from a 10-minute transmission scan with rotating germanium-68 rods and a corresponding 60-minute blank scan. Images were reconstructed into 2.34x2.34x4.25 mm voxels using the PROMIS 3D filtered back projection algorithm,[2] with corrections applied for randoms, dead time, normalisation, scatter, attenuation, and sensitivity. Emission images were smoothed using an isotropic 4 mm Gaussian filter, and subsequently, parametric maps of cerebral blood flow (CBF), blood volume (CBV), oxygen metabolism $CMRO_2$, and oxygen extraction fraction (OEF) were calculated by inputting image and arterial activity concentration measurements into standard models.[3,4] We used a blood-brain partition coefficient for ^{15}O water of 0.95 based upon the previous in vitro data[5] and a small to large vessel haematocrit ratio of 0.85.[6] Parametric PET images were coregistered to anatomy using structural magnetic resonance imaging (MRI) imaging or x-ray computed tomography (CT).

Image analysis

Imaging data were analysed using custom-designed automated software (PETAn)[7] incorporating elements of several software packages, including Statistical Parametric Mapping (SPM, Wellcome Department of Imaging Neuroscience, University College London), Matlab (MathWorks, Inc) and Analyze (AnalyzeDirect, Inc). Structural data (CT or T1-weighted MRI) were edited to extract a mask that identified brain tissue voxels and excluded extracranial tissue, cerebrospinal fluid, and extraaxial hematomas, and registered with PET. We used the brain mask to calculate CBF, $CMRO_2$, OEF, and CBV across the whole brain excluding extracranial tissue. All imaging data were subsequently spatially normalised to a template image using SPM and a region of interest (ROI) based analysis performed using 14 ROIs covering the whole brain with the exclusion of the brain stem (bilateral medial frontal, lateral frontal, temporal, parietal, occipital, deep gray matter and the cerebellum) as previously described (eFigure 2).[8] The ROI template was applied to the masked CBF, CBV, $CMRO_2$, and OEF images and the physiologic parameters were expressed as an average for all brain tissue within each of the ROIs. To limit regional analyses to viable brain, regions with obvious lesions were manually excluded using registered CT and MRI.

We estimated an individualised voxel-based critical OEF threshold (OEF_{crit}),[9,10] which equated to a cerebral venous oxygen content (CvO_2) of 3.5 mL/100mL,[11,12] for each subject as follows:

$$OEF_{crit} = \frac{(CaO_2 - 3.5)}{CaO_2}$$

where,

$$CaO_2 = 1.34\mathrm{Hb.SaO_2} + 0.0225\mathrm{PaO_2}$$

© 2020 Yoann Launey et al. *JAMA Neurology.*

CaO_2 is arterial oxygen content, Hb is the haemoglobin concentration in g/100mL, SaO_2 is the fractional arterial oxygen saturation and PaO_2 is the arterial partial pressure of oxygen (kPa).

© 2020 Yoann Launey et al. *JAMA Neurology.*

eReferences

1 Albeck MJ, Børgesen SE, Gjerris F, Schmidt JF, Sørensen PS. Intracranial pressure and cerebrospinal fluid outflow conductance in healthy subjects. *J Neurosurg* 1991; 74(4): 597-600.

2 Kinahan PE, Rogers JG. Analytic 3D image reconstruction using all detected events. *IEEE Trans Nucl Sci* 1989; 36: 964–8.

3 Frackowiak RS, Lenzi GL, Jones T, Heather JD. Quantitative measurement of regional cerebral blood flow and oxygen metabolism in man using 15O and positron emission tomography: theory, procedure, and normal values. *J Comput Assist Tomogr* 1980; 4: 727–36.

4 Lammertsma AA, Baron JC, Jones T. Correction for intravascular activity in the oxygen-15 steady-state technique is independent of the regional hematocrit. *J Cereb Blood Flow Metab* 1987; 7: 372–4.

5 Herscovitch P, Raichle ME. What is the correct value for the brain--blood partition coefficient for water? *J Cereb Blood Flow Metab* 1985; 5: 65–9.

6 Phelps ME, Huang SC, Hoffman EJ, Kuhl DE. Validation of tomographic measurement of cerebral blood volume with C-11-labeled carboxyhemoglobin. *J Nucl Med* 1979; 20: 328–34.

7 Smielewski P, Coles JP, Fryer TD, Minhas PS, Menon DK, Pickard JD. Integrated image analysis solutions for PET datasets in damaged brain. *J Clin Monit Comput* 2002; 17: 427–40.

8 Coles JP, Fryer TD, Coleman MR, *et al.* Hyperventilation following head injury: effect on ischemic burden and cerebral oxidative metabolism. *Crit Care Med* 2007; 35: 568–78.

9 Coles JP, Fryer TD, Smielewski P, et al. Defining ischemic burden after traumatic brain injury using 15O PET imaging of cerebral physiology. *J Cereb Blood Flow Metab* 2004; 24(2): 191-201.

10 Coles JP, Fryer TD, Smielewski P, et al. Incidence and mechanisms of cerebral ischemia in early clinical head injury. *J Cereb Blood Flow Metab* 2004; 24(2): 202-11.

11 Powers WJ, Grubb RL Jr., Darriet D, et al. Cerebral blood flow and cerebral metabolic rate of oxygen requirements for cerebral function and viability in humans. *J Cereb Blood Flow Metab* 1985; 5: 600–608.

12 Yundt KD, Diringer MN. The use of hyperventilation and its impact on cerebral ischemia in the treatment of traumatic brain injury. *Crit Care Clin* 1997; 13: 163–184.

© 2020 Yoann Launey et al. *JAMA Neurology.*

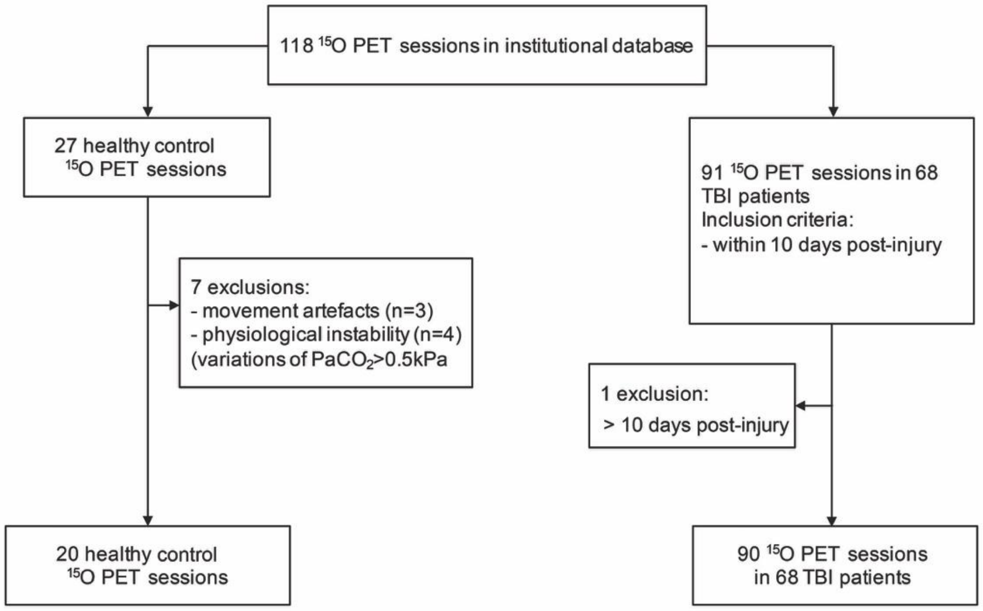

eFigure 1: Flowchart of oxygen-15 positron emission tomography data

^{15}O PET, oxygen-15 positron emission tomography; TBI, traumatic brain injury.

© 2020 Yoann Launey et al. *JAMA Neurology.*

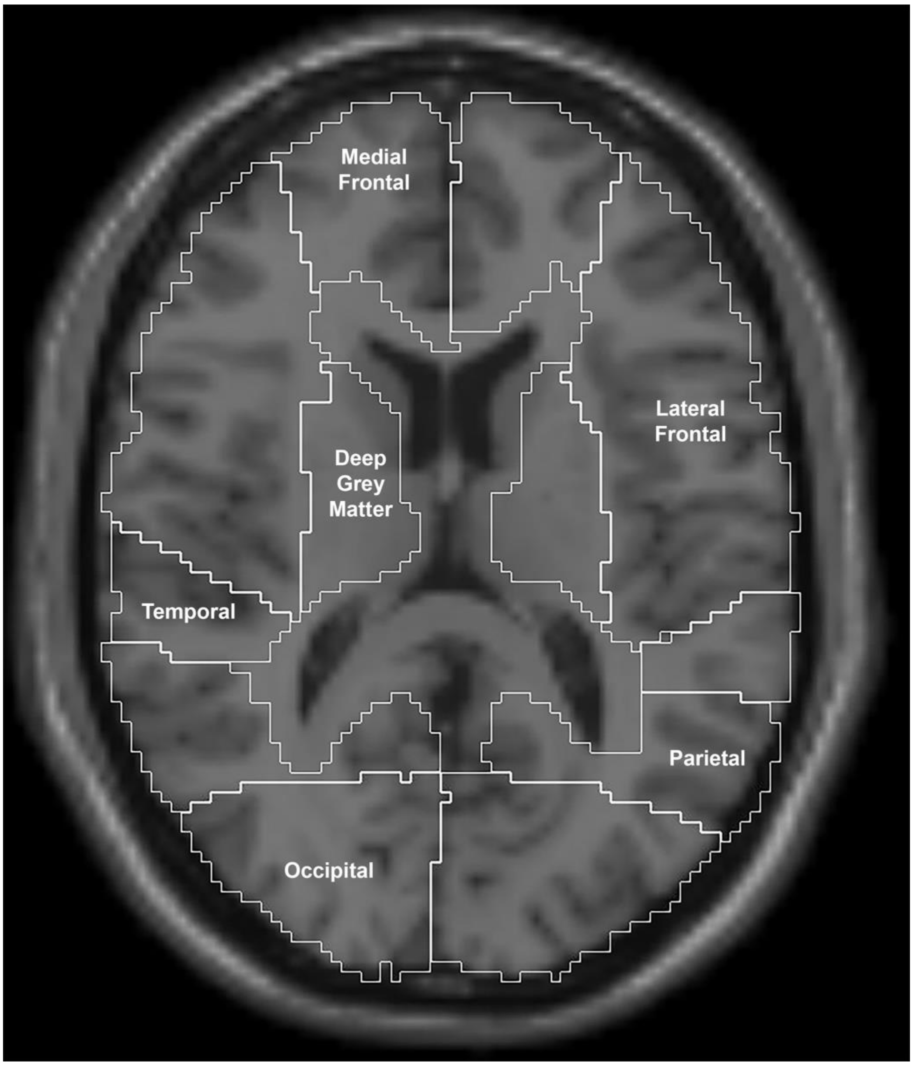

eFigure 2: Region of interest map

The region of interest (ROI) map is shown using a single slice from a reference T1 weighted magnetic resonance image. The regions shown are the right and left medial frontal, lateral frontal, temporal, parietal, occipital and deep grey matter. Not shown on this slice are the right and left cerebellar regions.

© 2020 Yoann Launey et al. *JAMA Neurology.*

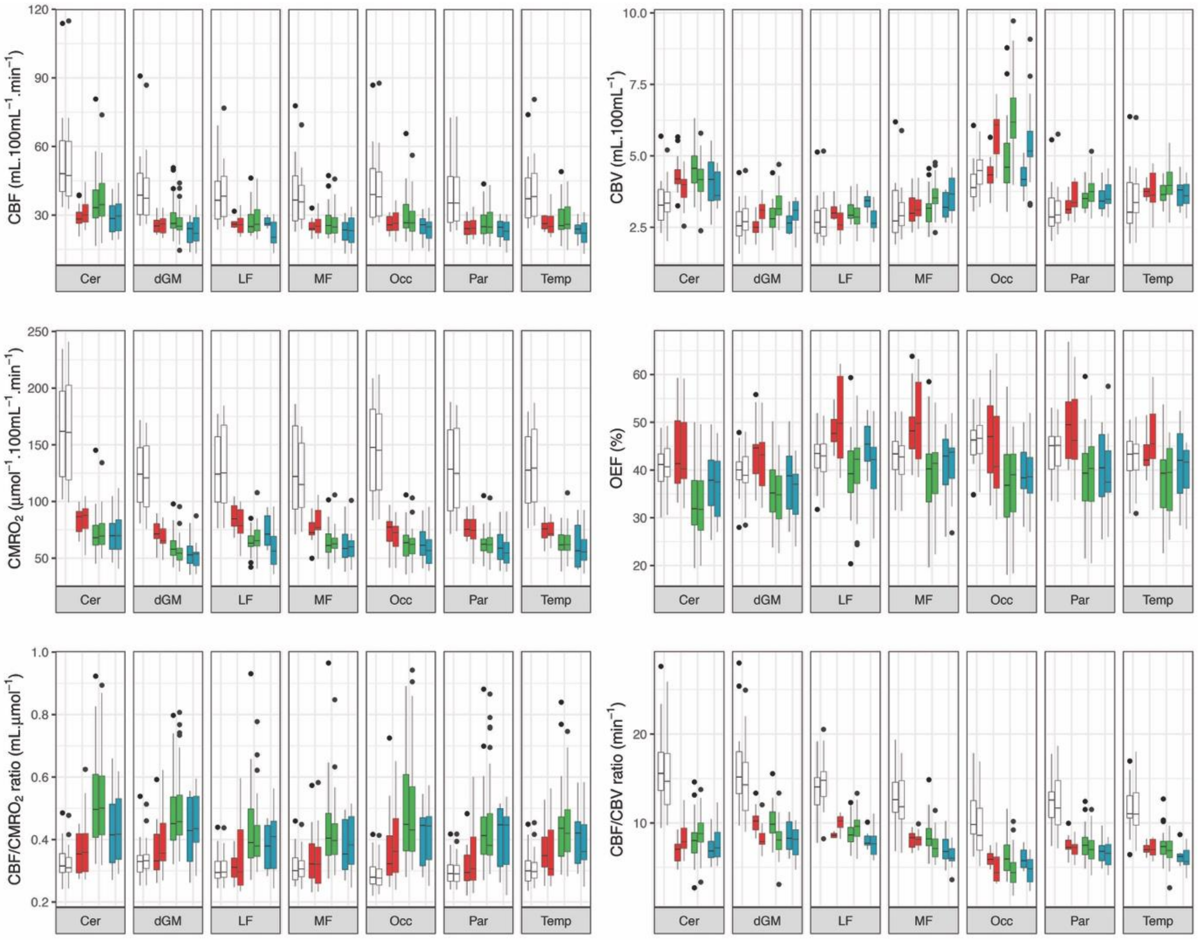

eFigure 3: Regional variation in physiology after traumatic brain injury

Box and whisker plots of physiology across left and right cerebellar (Cer), deep grey matter (dGM), lateral frontal (LF), medial frontal (MF), occipital (Occ), parietal (Par) and temporal (Temp) regions. Data shown are left followed by right region of interest (ROI) for each region from healthy control participants (white), and in patients after traumatic brain injury within 24 hours (Early; red), days 2 – 5 (Intermediate; green) and days 6 – 10 (Late, blue) post injury. The horizontal line within each box denotes the median value, the lower and upper boundaries the 25th and 75th centile, the vertical lines the 10th and 90th centile, and the closed circles outlying data points.

© 2020 Yoann Launey et al. *JAMA Neurology.*

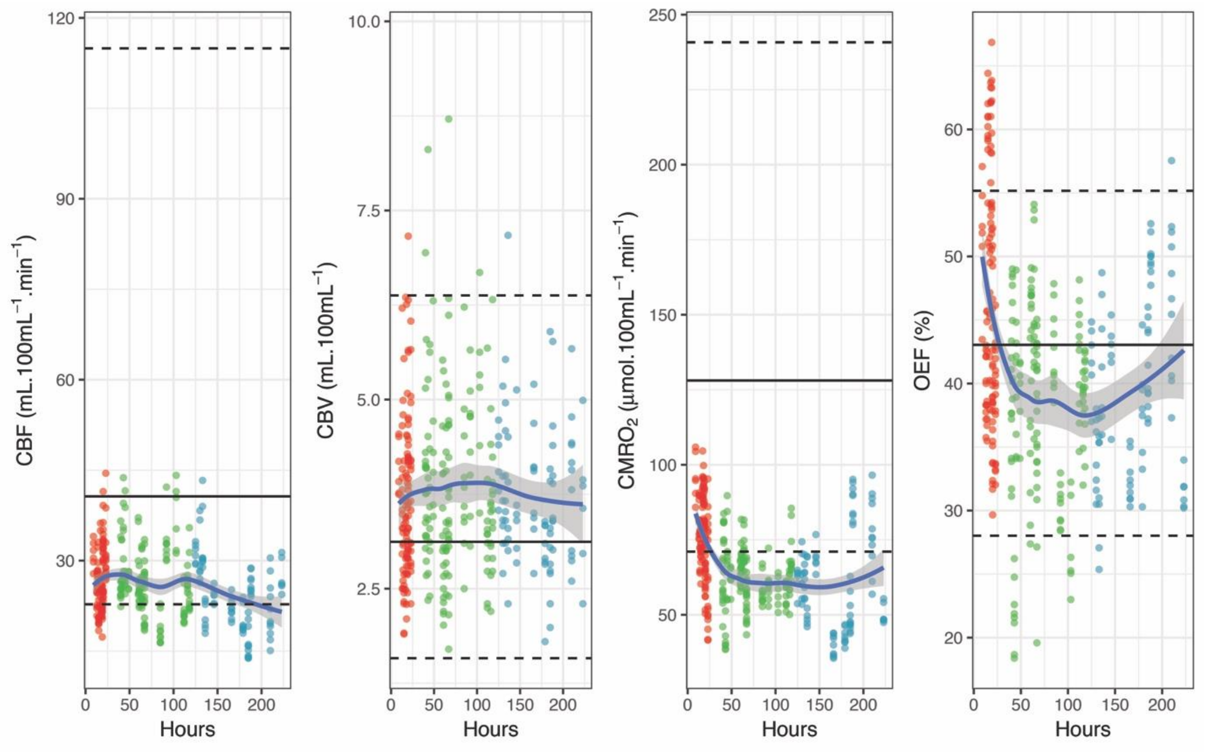

eFigure 4: Spatial and temporal pattern of regional physiological derangements in serial data obtained in patients after traumatic brain injury

Individual regional values for cerebral blood flow (CBF), blood volume (CBV), oxygen metabolism ($CMRO_2$) and oxygen extraction fraction (OEF) in 17 patients with traumatic brain injury plotted against time post injury (red, within 24 hours (n = 12, early); green, day 2 – 5 (n = 15, intermediate); blue, day 6 – 10 (n = 10, late)). The fitted light blue lines represent modelling of the relationship between each parameter and hours post injury using locally weighted scatterplot smoothing (LOWESS), with the 95% confidence interval shown in grey. The solid and dashed black lines represent the median and the full range of values for healthy control participants, respectively.

© 2020 Yoann Launey et al. *JAMA Neurology.*

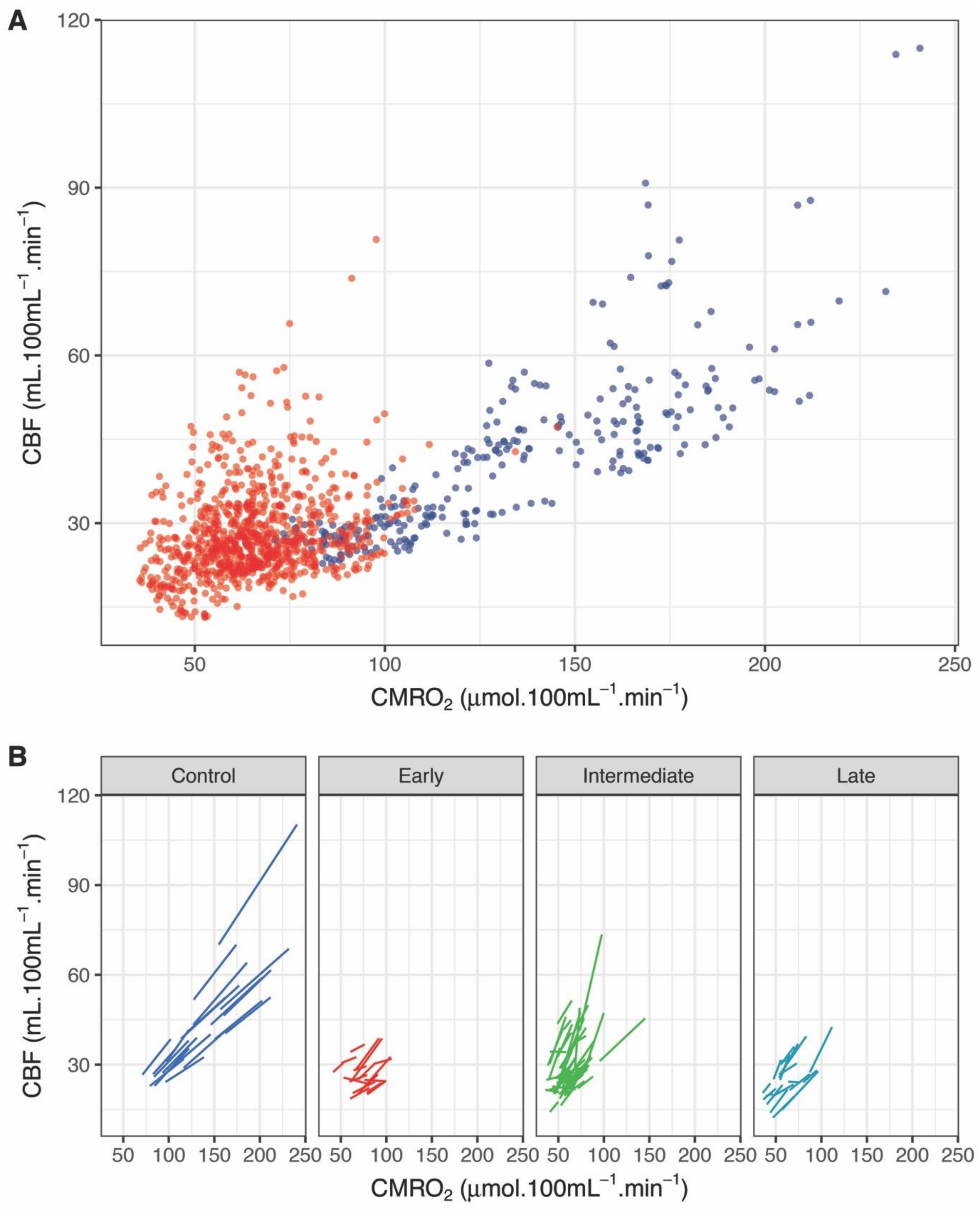

eFigure 5: Flow metabolism coupling

A. Scatterplot of association between cerebral blood flow (CBF) and cerebral oxygen metabolism ($CMRO_2$) for patients with traumatic brain injury (red circles) and healthy control participants (blue circles).

B. To illustrate the relationship within participants, region of interest (ROI) data from each individual subject are fitted with a linear regression line and shown for healthy control participants (Control), and in patients after traumatic brain injury within 24 hours (Early), days 2 – 5 (Intermediate) and days 6 – 10 (Late) post injury.

© 2020 Yoann Launey et al. *JAMA Neurology.*

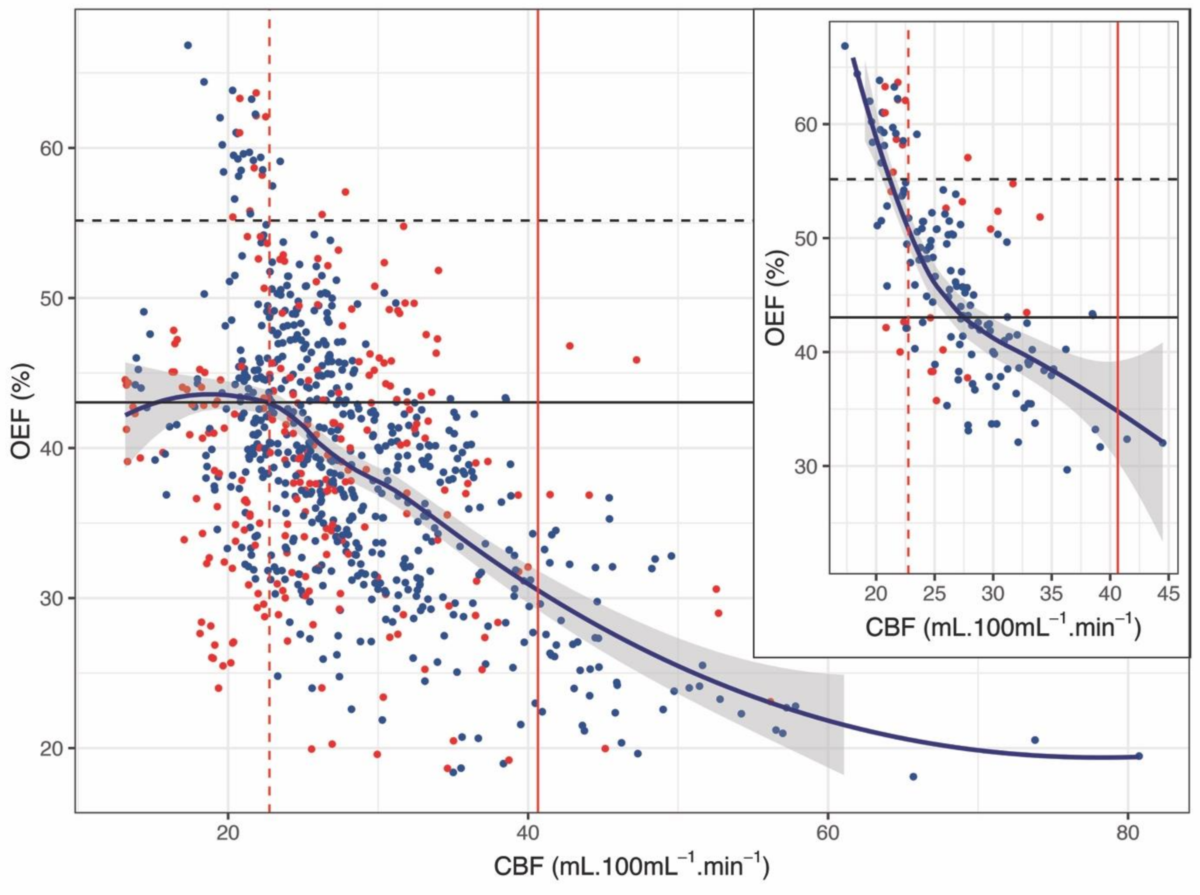

eFigure 6: Scatterplots of association between oxygen extraction fraction and cerebral blood flow in patients after traumatic brain injury

The association between regional oxygen extraction fraction (OEF) and cerebral blood flow (CBF) for the entire traumatic brain injury cohort is shown in the main panel. The regions from patients with intracranial pressure (ICP) > 20mmHg are shown in red, while those with ICP ≤ 20mmHg are shown in blue. The fitted blue line is the non-linear LOWESS regression model with 95% confidence interval shown in grey. The solid and dashed black lines represent the median and maximum OEF in healthy control participants, respectively. The solid and dashed red lines represent the median and minimum CBF in healthy control participants, respectively. **Insert**: Non-linear association between OEF and CBF within 24 hours of injury; the fitted blue line is the LOWESS regression model with 95% confidence interval shown in grey.

© 2020 Yoann Launey et al. *JAMA Neurology.*

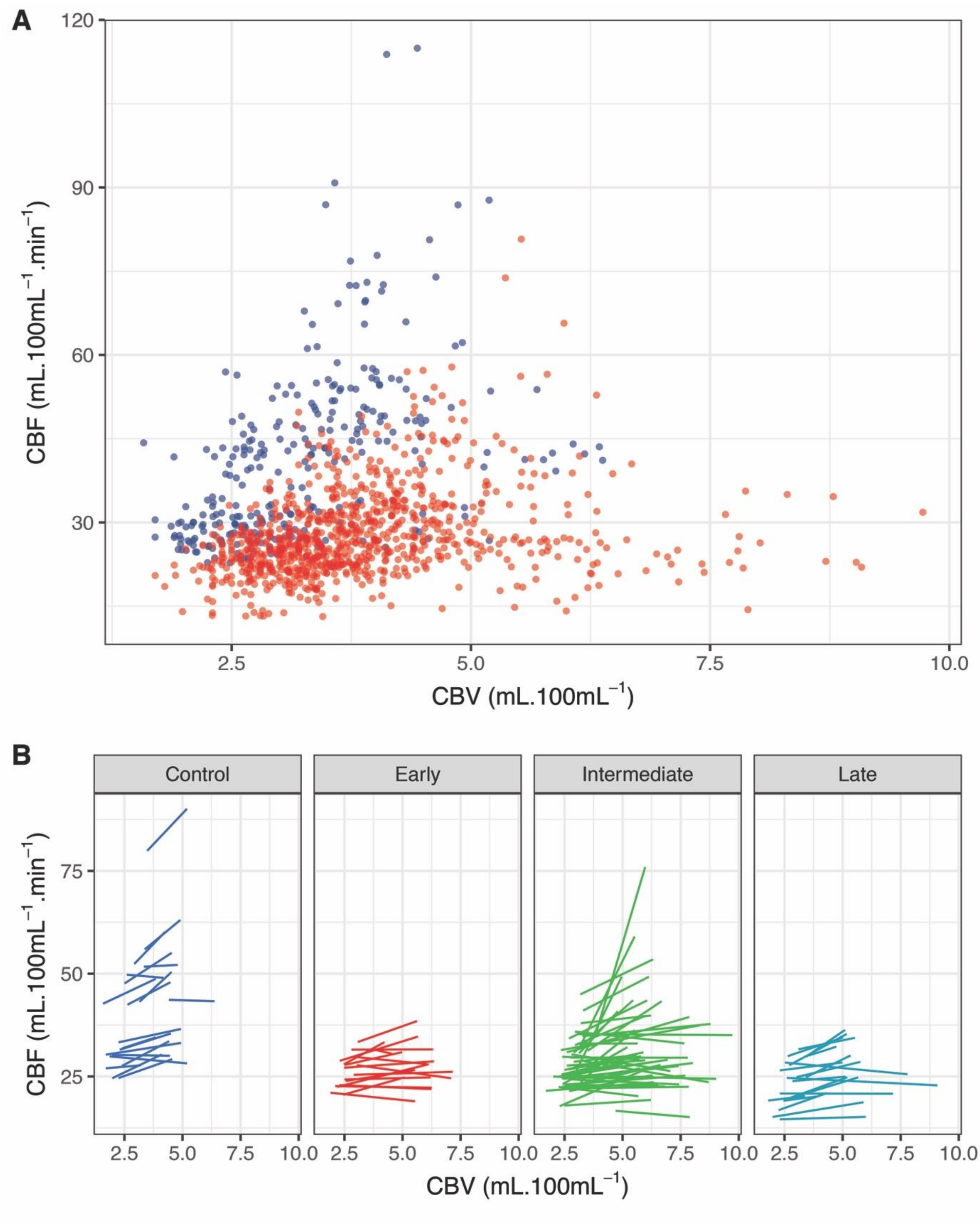

eFigure 7: Association between cerebral blood flow and blood volume

A. Scatterplot of the association between cerebral blood flow (CBF) and cerebral blood volume (CBV) for patients with traumatic brain injury (red circles) and healthy control participants (blue circles).

B. To illustrate the relationship within participants, the region of interest (ROI) data from each subject are fitted with a linear regression line and shown for healthy control participants (Control), and in patients after traumatic brain injury within 24 hours (Early), days 1 – 5 (Intermediate) and days 5 – 10 (Late) post injury.

© 2020 Yoann Launey et al. *JAMA Neurology.*

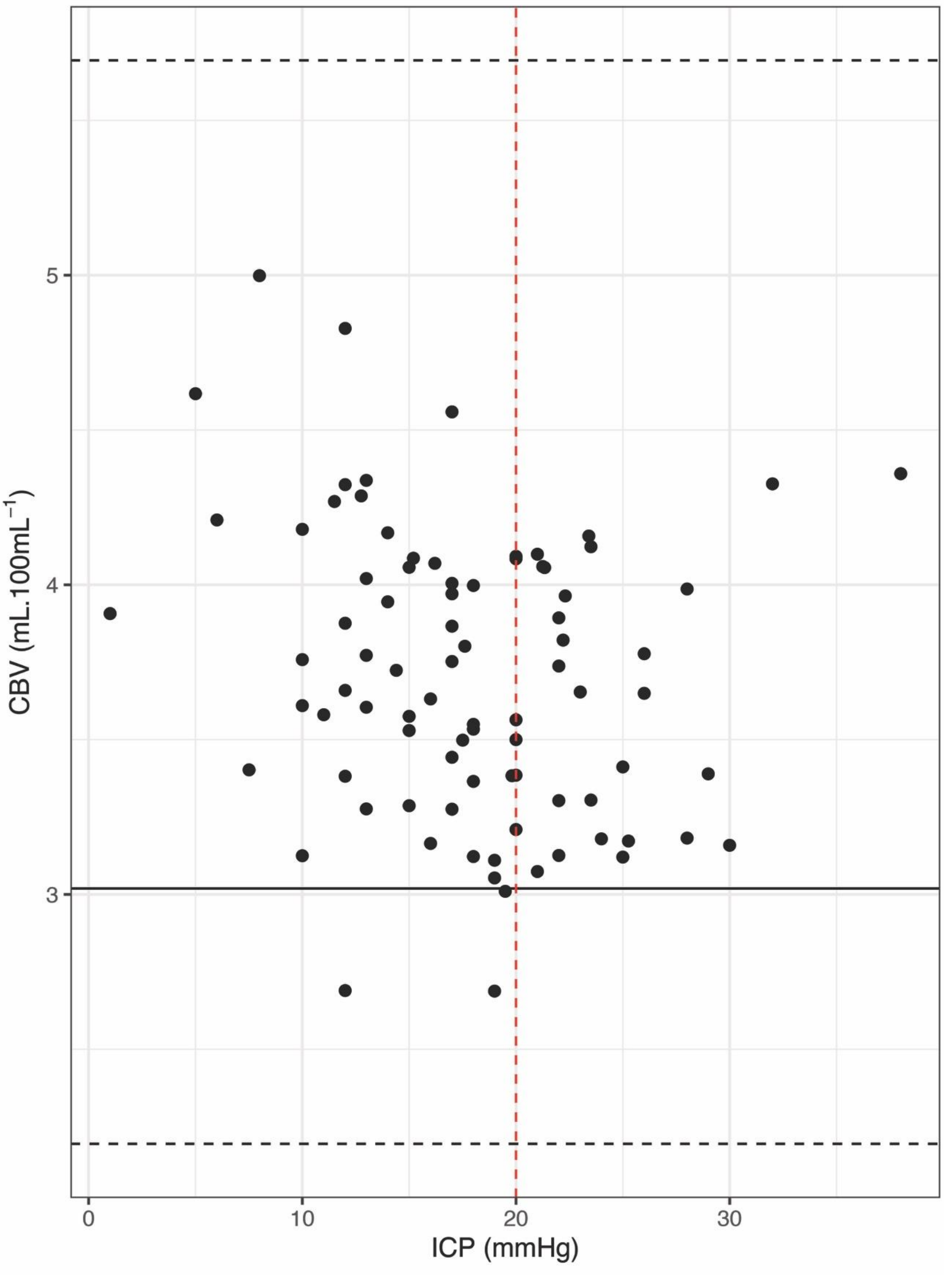

eFigure 8: Intracranial hypertension and cerebral blood volume

Scatterplot of the association between cerebral blood volume (CBV) and intracranial pressure (ICP) for patients with traumatic brain injury across all time points. The solid and dashed black lines represent the median and full range of CBV values in healthy control participants, respectively. The dashed red line marks the threshold for raised ICP at 20mmHg.

© 2020 Yoann Launey et al. *JAMA Neurology.*

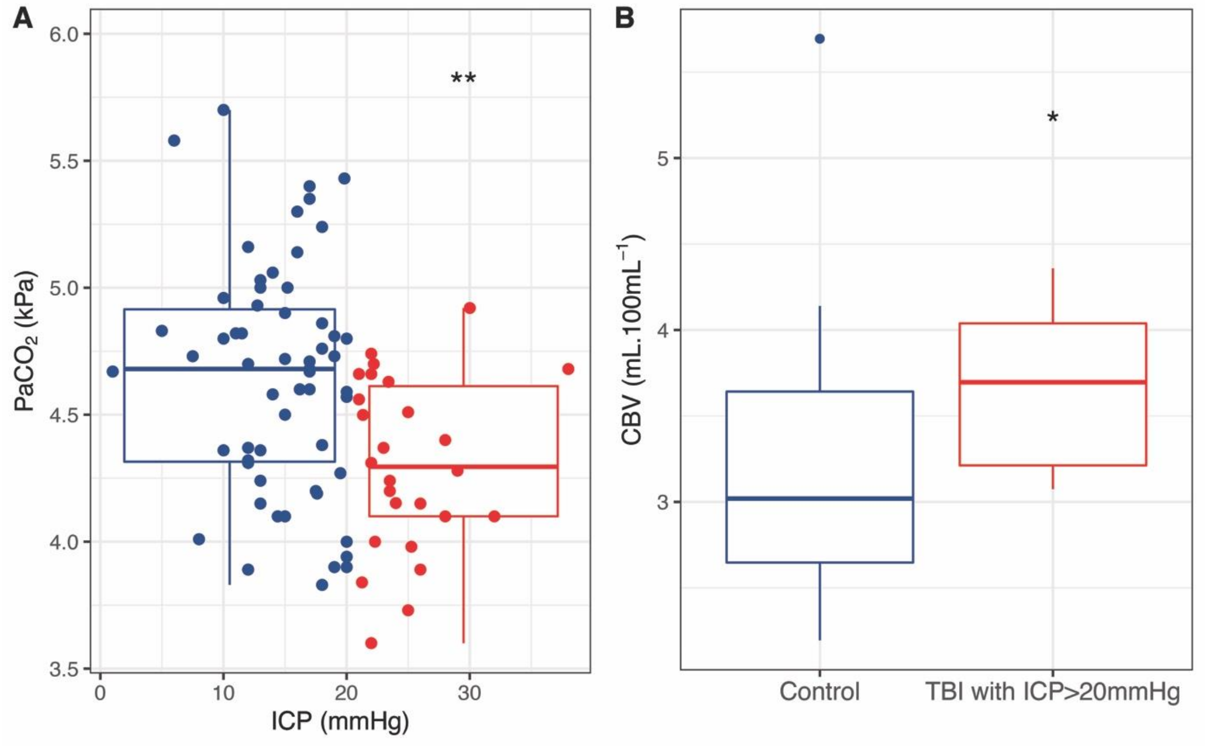

eFigure 9: Intracranial hypertension and hyperventilation

A. Scatterplot of the association between partial pressure of arterial carbon dioxide ($PaCO_2$) and intracranial pressure (ICP) for patients after traumatic brain injury during ^{15}oxygen positron emission tomography. Patients with ICP $\leq$ 20 and > 20mmHg are shown in blue and red respectively, with the data for the two groups also displayed as box and whisker plots. The horizontal line within each box denotes the median value, the lower and upper boundaries the 25th and 75th centile and the vertical lines the 10th and 90th centile.

B. Box and whisker plots of cerebral blood volume (CBV) for healthy control participants (blue) and patients after traumatic brain injury with ICP > 20mmHg, shown in (red).

*p-value = 0·002, **p-value = 0.001, Mann-Whitney test.

© 2020 Yoann Launey et al. *JAMA Neurology.*

A

	CBF			*CBV*			*$CMRO_2$*			*OEF*		
	**HC**	**TBI**	**p-value**	**HC**	**TBI**	**p-value**	**HC**	**TBI**	**p-value**	**HC**	**TBI**	**p-value**
**Cereb L**	48 (40–63)	32 (27–39)	**$<10^{-4}$**	3.3 (2.8–3.8)	4.5 (4.0–4.9)	**$<10^{-4}$**	162 (122–197)	71 (62–83)	**$<10^{-4}$**	41 (38–43)	35 (29–40)	0.005
**Cereb R**	47 (38–62)	33 (28–41)	**$<10^{-4}$**	3.3 (3.0–3.7)	4.0 (3.6–4.5)	**$<10^{-3}$**	161 (119–203)	72 (63–83)	**$<10^{-4}$**	41 (39–45)	34 (30–40)	0.001
**DGM L**	39 (30–48)	26 (24–29)	**$<10^{-4}$**	2.6 (2.2–3.0)	2.7 (2.4–3.1)	0.267	124 (99–148)	60 (53–68)	**$<10^{-4}$**	40 (38–42)	38 (32–42)	0.074
**DGM R**	37 (30–46)	25 (22–29)	**$<10^{-4}$**	2.7 (2.4–3.2)	3.1 (2.9–3.5)	0.002	121 (95–149)	55 (49–64)	**$<10^{-4}$**	39 (37–43)	36 (31–40)	0.020
**LF L**	37 (28–45)	25 (23–29)	**$<10^{-4}$**	2.7 (2.3–3.1)	3.0 (2.8–3.4)	0.010	124 (100–157)	66 (61–79)	**$<10^{-4}$**	44 (40–45)	43 (38–46)	0.770
**LF R**	38 (29–47)	25 (22–29)	**$<10^{-4}$**	2.5 (2.2–3.2)	2.7 (2.5–3.1)	0.231	125 (99–167)	67 (61–77)	**$<10^{-4}$**	43 (40–46)	42 (39–46)	0.873
**MF L**	37 (28–47)	25 (22–29)	**$<10^{-4}$**	2.7 (2.3–3.8)	3.1 (2.7–3.5)	0.155	122 (93–167)	64 (55–74)	**$<10^{-4}$**	43 (40–46)	42 (36–48)	0.630
**MF R**	36 (28–43)	25 (22–29)	**$<10^{-4}$**	2.9 (2.6–3.4)	3.5 (3.1–3.9)	0.003	115 (95–152)	65 (59–75)	**$<10^{-4}$**	43 (39–45)	42 (38–46)	1
**Occip L**	39 (29–50)	26 (24–31)	**$<10^{-4}$**	3.9 (3.6–4.4)	4.4 (4.0–5.0)	0.013	148 (109–181)	64 (54–74)	**$<10^{-4}$**	46 (43–48)	38 (33–46)	0.001
**Occip R**	38 (30–49)	26 (23–31)	**$<10^{-4}$**	4.5 (4.0–4.9)	5.9 (5.1–6.6)	**$<10^{-4}$**	145 (110–177)	63 (53–72)	**$<10^{-4}$**	47 (44–49)	39 (32–44)	**$<10^{-3}$**
**Par L**	35 (27–47)	24 (22–28)	**$<10^{-4}$**	2.9 (2.5–3.5)	3.4 (3.1–3.7)	0.005	129 (92–163)	63 (57–71)	**$<10^{-4}$**	45 (40–47)	41 (36–47)	0.103
**Par R**	35 (27–47)	24 (21–30)	**$<10^{-4}$**	2.9 (2.7–3.4)	3.6 (3.3–4.0)	**$<10^{-4}$**	125 (94–165)	64 (55–70)	**$<10^{-4}$**	45 (41–47)	42 (35–45)	0.064
**Temp L**	37 (29–46)	25 (23–30)	**$<10^{-4}$**	3.0 (2.6–4.1)	3.7 (3.4–3.9)	0.116	128 (99–157)	64 (57–75)	**$<10^{-4}$**	43 (40–46)	40 (34–44)	0.105
**Temp R**	38 (30–48)	26 (22–31)	**$<10^{-4}$**	3.4 (3.0–4.0)	3.9 (3.5–4.3)	0.017	130 (100–164)	64 (56–71)	**$<10^{-4}$**	44 (40–45)	41 (35–45)	0.136

B

	$CBF/CMRO_2$ ratio			*CBF/CBV ratio*			*CBF/CBV/CPP*		
	**HC**	**TBI**	**p-value**	**HC**	**TBI**	**p value**	**HC**	**TBI**	**p-value**
**Cereb L**	0.32 (0.29-0.36)	0.45 (0.37-0.55)	**$<10^{-4}$**	15.6 (13.7-17.9)	7.4 (6.5-7.4)	**$<10^{-4}$**	0.23 (0.19-0.26)	0.10 (0.08-0.12)	**$<10^{-4}$**
**Cereb R**	0.31 (0.30-0.34)	0.47 (0.37-0.57)	**$<10^{-4}$**	14.7 (12.1-17.8)	8.8 (7.2-8.8)	**$<10^{-4}$**	0.23 (0.17-0.25)	0.11 (0.10-0.13)	**$<10^{-4}$**
**DGM L**	0.33 (0.30-0.35)	0.43 (0.37-0.53)	**$<10^{-4}$**	15.2 (13.3-18.0)	10.0 (8.7-10.0)	**$<10^{-4}$**	0.23 (0.18-0.25)	0.13 (0.12-0.15)	**$<10^{-4}$**
**DGM R**	0.33 (0.31-0.35)	0.44 (0.38-0.54)	**$<10^{-4}$**	14.3 (11.4-16.8)	8.1 (7.1-8.1)	**$<10^{-4}$**	0.21 (0.18-0.23)	0.11 (0.09-0.13)	**$<10^{-4}$**
**LF L**	0.29 (0.28-0.33)	0.38 (0.32-0.45)	**$<10^{-3}$**	14.0 (12.1-15.1)	8.5 (7.8-8.5)	**$<10^{-4}$**	0.20 (0.18-0.22)	0.11 (0.10-0.13)	**$<10^{-4}$**
**LF R**	0.30 (0.28-0.33)	0.37 (0.32-0.45)	$<10^{-3}$	14.8 (13.0-15.8)	9.5 (8.2-9.5)	**$<10^{-4}$**	0.21 (0.18-0.23)	0.13 (0.11-0.14)	**$<10^{-4}$**
**MF L**	0.30 (0.27-0.34)	0.38 (0.32-0.47)	**$<10^{-3}$**	12.6 (11.2-14.6)	8.1 (6.9-8.1)	**$<10^{-4}$**	0.18 (0.16-0.21)	0.11 (0.09-0.13)	**$<10^{-4}$**
**MF R**	0.31 (0.28-0.33)	0.38 (0.33-0.45)	**$<10^{-3}$**	11.8 (10.5-14.8)	7.2 (6.2-7.2)	**$<10^{-4}$**	0.18 (0.15-0.21)	0.10 (0.08-0.11)	**$<10^{-4}$**
**Occip L**	0.28 (0.26-0.31)	0.42 (0.33-0.52)	**$<10^{-4}$**	9.8 (8.8-12.5)	5.9 (5.0-5.9)	**$<10^{-4}$**	0.15 (0.12-0.16)	0.08 (0.06-0.09)	**$<10^{-4}$**
**Occip R**	0.28 (0.25-0.31)	0.43 (0.34-0.50)	**$<10^{-4}$**	8.6 (6.9-11.7)	4.5 (3.3-4.5)	**$<10^{-4}$**	0.12 (0.10-0.16)	0.06 (0.04-0.08)	**$<10^{-4}$**
**Par L**	0.29 (0.27-0.32)	0.38 (0.33-0.48)	**$<10^{-4}$**	12.6 (10.6-13.5)	7.2 (6.3-7.2)	**$<10^{-4}$**	0.18 (0.14-0.20)	0.09 (0.08-0.11)	**$<10^{-4}$**
**Par R**	0.29 (0.27-0.32)	0.38 (0.34-0.48)	**$<10^{-4}$**	11.7 (9.8-14.8)	6.8 (5.9-6.8)	**$<10^{-4}$**	0.17 (0.14-0.20)	0.09 (0.08-0.11)	**$<10^{-4}$**
**Temp L**	0.30 (0.27-0.33)	0.42 (0.35-0.46)	**$<10^{-4}$**	11.0 (10.6-13.1)	7.0 (6.2-7.0)	**$<10^{-4}$**	0.17 (0.14-0.20)	0.09 (0.08-0.11)	**$<10^{-4}$**
**Temp R**	0.30 (0.28-0.33)	0.40 (0.34-0.48)	**$<10^{-4}$**	11.0 (9.8-13.4)	6.8 (5.9-6.8)	**$<10^{-4}$**	0.17 (0.14-0.19)	0.09 (0.08-0.11)	**$<10^{-4}$**

© 2020 Yoann

eTable 1: Regional physiological derangements

Data shown are median (interquartile range) for healthy control participants (HC) and patients after traumatic brain injury (TBI) acquired at all time points from 14 regions of interest (ROI) covering the whole brain excluding the brain stem. Regional cerebral blood flow (CBF, ml/100ml/min), cerebral blood volume (CBV, ml/100ml), cerebral oxygen metabolism ($CMRO_2$, μmol/100ml/min) and oxygen extraction fraction (OEF, %) are shown in **A**, while CBF/$CMRO_2$, CBF/CBV and the cerebral perfusion pressure (CPP) indexed CBF/CBV are shown in **B**. Mann-Whitney tests were used for comparisons between all TBI patients and healthy control participants. Significant p-values that survive correction for multiple comparisons are highlighted in bold. Cereb: cerebellum. DGM: deep grey matter. LF: lateral frontal. MF: medial frontal. Occip: occipital. Par: parietal. Temp: temporal. L: left. Right: right.

© 2020 Yoann Launey et al. *JAMA Neurology.*

A

	CBF					CBV					$CMRO_2$					OEF				
	**HC**	**Early**	**Inter**	**Late**	**p-value**	**HC**	**Early**	**Inter**	**Late**	**p-value**	**HC**	**Early**	**Inter**	**Late**	**p-value**	**HC**	**Early**	**Inter**	**Late**	**p-value**
Cereb L	48 (40-63)	**28**[A] (26-31)	**33**[A] (29-41)	**29**[A] (23-34)	$<10^{-4}$	3.3 (2.8-3.8)	4.2 (4.0-4.5)	**4.6**[A] (4.0-5.0)	4.2 (3.4-4.8)	$<10^{-4}$	162 (122-197)	87 (74-91)	**68**[A] (62-79)	**70**[A] (58-81)	$<10^{-4}$	41 (38-43)	**41**[B] (37-50)	**32**[A] (29-38)	38 (33-42)	$<10^{-4}$
Cereb R	47 (38-62)	**30**[A] (27-35)	35 (30-44)	**30**[A] (23-35)	$<10^{-4}$	3.3 (3.0-3.7)	4.0 (3.5-4.2)	**4.2**[A] (3.7-4.5)	3.6 (3.4-4.4)	$<10^{-3}$	161 (119-203)	88 (76-94)	**70**[A] (62-81)	**70**[A] (58-84)	$<10^{-4}$	41 (39-45)	**40**[B] (38-50)	**32**[A] (27-38)	38 (30-42)	$<10^{-4}$
DGM L	39 (30-48)	**25**[A] (23-28)	**26**[A] (24-31)	**24**[A] (18-27)	$<10^{-4}$	2.6 (2.2-3.0)	2.5 (2.3-2.7)	2.8 (2.5-3.2)	2.7 (2.3-2.9)	0.099	124 (99-148)	71 (67-80)	**58**[A] (53-66)	**53**[A] (45-61)	$<10^{-4}$	40 (38-42)	45 (39-45)	35 (31-40)	39 (32-43)	$<10^{-3}$
DGM R	37 (30-46)	26 (22-28)	**25**[A] (24-29)	**22**[A] (19-29)	$<10^{-4}$	2.7 (2.4-3.2)	3.1 (2.8-3.3)	3.2 (2.9-3.6)	3.1 (2.8-3.4)	0.006	121 (95-149)	65 (63-76)	**55**[A] (48-59)	**54**[A] (44-55)	$<10^{-4}$	39 (37-43)	43 (37-46)	35 (30-39)	37 (31-39)	$<10^{-3}$
LF L	37 (28-45)	26.0 (25-27)	25.0 (23-30)	26 (26-29)	0.001	2.7 (2.3-3.1)	3.0 (2.8-3.2)	2.9 (2.8-3.3)	3.4 (3.2-3.6)	0.044	124 (100-157)	85 (78-96)	**63**[A] (60-70)	71 (61-88)	$<10^{-4}$	44 (40-45)	48 (46-51)	39 (35-44)	45 (42-49)	0.009
LF R	38 (29-47)	26 (22-29)	26 (23-32)	**20**[A] (18-27)	$<10^{-4}$	2.5 (2.2-3.2)	2.6 (2.4-3.0)	2.9 (2.6-3.2)	2.6 (2.5-3.1)	0.258	125 (99-167)	79 (72-93)	**66**[A] (61-75)	**56**[A] (45-69)	$<10^{-4}$	43 (40-46)	50 (43-60)	42 (37-45)	42 (36-45)	0.026
MF L	37 (28-47)	24 (23-27)	25 (22-30)	24 (19-27)	$<10^{-3}$	2.7 (2.3-3.8)	3.0 (2.7-3.5)	3.2 (2.7-3.3)	3.2 (2.9-3.7)	0.475	122 (93-167)	73 (71-82)	**61**[A] (55-71)	**59**[A] (51-71)	$<10^{-4}$	43 (40-46)	48 (44-51)	40 (33-43)	43 (37-47)	0.004
MF R	36 (28-43)	25 (22-28)	25 (22-29)	23 (18-29)	$<10^{-3}$	2.9 (2.6-3.4)	3.1 (2.9-3.5)	3.5 (3.3-3.8)	3.7 (3.1-4.2)	0.006	115 (95-152)	77 (75-92)	**63**[A] (59-68)	**60**[A] (51-66)	$<10^{-4}$	43 (39-45)	50 (42-58)	41 (35-44)	44 (38-45)	0.010
Occip L	39 (29-50)	26 (23-29)	27 (24-35)	**26**[A] (22-28)	$<10^{-4}$	3.9 (3.6-4.4)	4.3 (4.0-4.6)	4.6 (4.1-5.4)	4.2 (3.9-4.6)	0.024	148 (109-181)	78 (65-82)	**64**[A] (52-70)	**61**[A] (53-74)	$<10^{-4}$	46 (43-48)	47 (39-54)	**37**[A] (30-43)	38 (36-44)	$<10^{-4}$
Occip R	38 (30-49)	26 (23-31)	**27**[A] (23-32)	**25**[A] (20-27)	$<10^{-4}$	4.5 (4.0-4.9)	6.1 (5.1-6.3)	**6.2**[A] (5.6-7.0)	5.2 (5.0-5.9)	$<10^{-4}$	145 (110-177)	73 (60-78)	**62**[A] (54-68)	**57**[A] (45-66)	$<10^{-4}$	47 (44-49)	41 (36-51)	**39**[A] (31-43)	39 (35-43)	$<10^{-3}$
Par L	35 (27-47)	24 (21-27)	25 (22-30)	25 (20-27)	$<10^{-3}$	2.9 (2.5-3.5)	3.1 (3.0-3.4)	3.5 (3.2-3.7)	3.4 (3.2-3.8)	0.012	129 (92-163)	76 (69-84)	**62**[A] (57-67)	**59**[A] (49-71)	$<10^{-4}$	45 (40-47)	50 (42-54)	39 (34-44)	41 (34-47)	0.001
Par R	35 (27-47)	24 (22-28)	25 (22-31)	**23**[A] (19-27)	$<10^{-4}$	2.9 (2.7-3.4)	3.4 (3.2-4.1)	**3.7**[A] (3.4-4.0)	3.5 (3.3-4.0)	$<10^{-3}$	125 (94-165)	75 (67-84)	**62**[A] (55-68)	**55**[A] (46-64)	$<10^{-4}$	45 (41-47)	46 (42-55)	40 (33-45)	38 (35-44)	0.004
Temp L	37 (29-46)	26 (24-30)	25 (24-33)	24 (22-26)	$<10^{-3}$	3.0 (2.6-4.1)	3.8 (3.6-3.9)	3.6 (3.4-4.0)	3.8 (3.3-4.0)	0.467	128 (99-157)	76 (68-81)	**62**[A] (57-71)	**56**[A] (42-79)	$<10^{-4}$	43 (40-46)	42 (41-45)	39 (32-42)	42 (35-48)	0.090
Temp R	38 (30-48)	25 (22-30)	26 (24-34)	**22**[A] (18-27)	$<10^{-4}$	3.4 (3.0-4.0)	3.6 (3.4-4.4)	4.0 (3.6-4.4)	3.6 (3.3-4.0)	0.028	130 (100-164)	71 (70-82)	**62**[A] (57-70)	**56**[A] (48-67)	$<10^{-4}$	44 (40-45)	46 (42-52)	40 (32-45)	42 (36-44)	0.069

© 2020 Yoann Launey et al. *JAMA Neurology.*

B

	CBF/CMRO₂ ratio					*CBF/CBV ratio*					*CBF/CBV/CPP*				
	HC	Early	Inter	Late	p-value	HC	Early	Inter	Late	p-value	HC	Early	Inter	Late	p-value
Cereb L	0.32 (0.29-0.36)	**0.35**[B] (0.29-0.42)	**0.50**[A] (0.41-0.61)	0.41 (0.33-0.51)	$<10^{-4}$	15.6 (13.7-17.9)	**7.0**[A] (5.7-7.6)	**8.0**[A] (6.6-8.9)	**6.8**[A] (6.1-7.9)	$<10^{-4}$	0.23 (0.19-0.26)	**0.09**[A] (0.08-0.10)	**0.11**[A] (0.09-0.13)	**0.09**[A] (0.08-0.11)	$<10^{-4}$
Cereb R	0.31 (0.30-0.34)	0.36 (0.30-0.42)	**0.50**[A] (0.41-0.60)	0.42 (0.34-0.53)	$<10^{-4}$	14.7 (12.1-17.8)	**7.9**[A] (7.1-9.4)	**8.8**[A] (7.8-10.0)	**7.2**[A] (6.2-8.9)	$<10^{-4}$	0.23 (0.17-0.25)	**0.11**[A] (0.10-0.12)	**0.12**[A] (0.10-0.14)	**0.10**[A] (0.08-0.11)	$<10^{-4}$
DGM L	0.33 (0.30-0.35)	0.33 (0.30-0.42)	**0.45**[A] (0.40-0.54)	0.43 (0.33-0.54)	$<10^{-4}$	15.2 (13.3-18.0)	10.2 (9.2-10.8)	**9.9**[A] (8.9-11.2)	**8.3**[A] (7.0-10.1)	$<10^{-4}$	0.23 (0.18-0.25)	**0.13**[A] (0.12-0.14)	**0.14**[A] (0.12-0.16)	**0.09**[A] (0.08-0.11)	$<10^{-4}$
DGM R	0.33 (0.31-0.35)	0.36 (0.33-0.45)	**0.46**[A] (0.41-0.54)	0.43 (0.39-0.54)	$<10^{-4}$	14.3 (11.4-16.8)	**7.9**[A] (7.6-8.8)	**8.1**[A] (7.0-9.0)	**8.2**[A] (6.4-9.2)	$<10^{-4}$	0.21 (0.18-0.23)	**0.11**[A] (0.10-0.12)	**0.11**[A] (0.09-0.13)	**0.10**[A] (0.08-0.12)	$<10^{-4}$
LF L	0.29 (0.28-0.33)	0.31 (0.28-0.34)	**0.39**[A] (0.34-0.50)	0.38 (0.31-0.45)	$<10^{-3}$	14.0 (12.1-15.1)	8.6 (8.4-8.8)	**8.6**[A] (7.9-9.5)	7.7 (7.5-8.5)	$<10^{-4}$	0.20 (0.18-0.22)	0.12 (0.11-0.12)	**0.11**[A] (0.10-0.14)	0.11 (0.10-0.11)	$<10^{-4}$
LF R	0.30 (0.28-0.33)	0.30 (0.25-0.43)	**0.38**[A] (0.35-0.45)	0.41 (0.31-0.46)	$<10^{-3}$	14.8 (13.0-15.8)	10.2 (9.5-10.7)	**9.6**[A] (8.5-10.3)	**7.7**[A] (6.5-8.5)	$<10^{-4}$	0.21 (0.18-0.23)	0.13 (0.13-0.15)	**0.13**[A] (0.11-0.14)	**0.10**[A] (0.08-0.11)	$<10^{-4}$
MF L	0.30 (0.27-0.34)	0.32 (0.28-0.39)	**0.40**[A] (0.35-0.48)	0.35 (0.30-0.46)	$<10^{-3}$	12.6 (11.2-14.6)	**8.3**[A] (7.3-8.8)	**8.2**[A] (7.4-9.4)	**6.8**[A] (6.0-8.2)	$<10^{-4}$	0.18 (0.16-0.21)	0.11 (0.10-0.13)	**0.11**[A] (0.09-0.13)	**0.09**[A] (0.07-0.10)	$<10^{-4}$
MF R	0.31 (0.28-0.33)	0.32 (0.26-0.38)	**0.40**[A] (0.35-0.45)	0.38 (0.32-0.47)	$<10^{-3}$	11.8 (10.5-14.8)	8.1 (7.5-8.5)	**7.1**[A] (6.2-8.2)	**6.0**[A] (5.7-7.1)	$<10^{-4}$	0.18 (0.15-0.21)	0.10 (0.10-0.12)	**0.09**[A] (0.08-0.12)	**0.07**[A] (0.07-0.09)	$<10^{-4}$
Occip L	0.28 (0.26-0.31)	0.32 (0.29-0.41)	**0.45**[A] (0.36-0.61)	0.44 (0.32-0.47)	$<10^{-4}$	9.8 (8.8-12.5)	**5.9**[A] (5.3-6.6)	**5.9**[A] (4.6-7.5)	**5.8**[A] (5.0-7.0)	$<10^{-4}$	0.15 (0.12-0.16)	0.08 (0.07-0.09)	**0.08**[A] (0.06-0.10)	0.07 (0.06-0.08)	$<10^{-4}$
Occip R	0.28 (0.25-0.31)	0.36 (0.29-0.47)	**0.43**[A] (0.36-0.57)	**0.44**[A] (0.34-0.47)	$<10^{-4}$	8.6 (6.9-11.7)	**4.4**[A] (3.5-6.2)	**4.4**[A] (3.3-5.5)	**4.8**[A] (3.4-5.9)	$<10^{-4}$	0.12 (0.10-0.16)	**0.06**[A] (0.05-0.09)	**0.06**[A] (0.04-0.08)	**0.06**[A] (0.04-0.07)	$<10^{-4}$
Par L	0.29 (0.27-0.32)	0.29 (0.27-0.35)	**0.41**[A] (0.35-0.47)	0.45 (0.31-0.50)	$<10^{-4}$	12.6 (10.6-13.5)	**7.2**[A] (7.1-8.1)	**7.5**[A] (6.4-8.4)	**6.8**[A] (5.8-7.5)	$<10^{-4}$	0.18 (0.14-0.20)	0.10 (0.09-0.11)	**0.10**[A] (0.09-0.12)	**0.08**[A] (0.08-0.10)	$<10^{-4}$
Par R	0.29 (0.27-0.32)	0.31 (0.27-0.41)	**0.38**[A] (0.35-0.48)	0.45 (0.32-0.47)	$<10^{-4}$	11.7 (9.8-14.8)	**7.2**[A] (6.5-7.6)	**7.0**[A] (5.9-8.0)	**6.6**[A] (5.3-7.6)	$<10^{-4}$	0.17 (0.14-0.20)	**0.09**[A] (0.08-0.11)	**0.09**[A] (0.08-0.11)	**0.07**[A] (0.06-0.09)	$<10^{-4}$
Temp L	0.30 (0.27-0.33)	0.35 (0.31-0.41)	**0.44**[A] (0.35-0.47)	0.42 (0.32-0.46)	$<10^{-3}$	11.0 (10.6-13.1)	7.0 (6.7-7.5)	**7.3**[A] (6.5-7.9)	**6.2**[A] (5.6-6.5)	$<10^{-4}$	0.17 (0.14-0.20)	0.10 (0.09-0.11)	**0.10**[A] (0.09-0.12)	**0.07**[A] (0.07-0.09)	$<10^{-4}$
Temp R	0.30 (0.28-0.33)	0.31 (0.29-0.43)	**0.42**[A] (0.36-0.50)	0.36 (0.34-0.45)	$<10^{-4}$	11.0 (9.8-13.4)	7.0 (6.4-8.2)	**6.9**[A] (6.1-8.0)	**5.7**[A] (5.3-6.9)	$<10^{-4}$	0.17 (0.14-0.19)	0.09 (0.09-0.11)	**0.09**[A] (0.08-0.11)	**0.07**[A] (0.06-0.09)	$<10^{-4}$

eTable 2: Temporal pattern of regional physiological derangements

Data shown are median (interquartile range) for healthy control participants (HC) and patients after traumatic brain injury (TBI) acquired within 24 hours (Early), between day 2 – 5 (Inter) and day 6 – 10 (Late) post injury from 14 regions of interest (ROI) covering the whole brain excluding the brain stem. Regional cerebral blood flow (CBF, ml/100ml/min), cerebral blood volume (CBV, ml/100ml), cerebral oxygen metabolism ($CMRO_2$, µmol/100ml/min) and oxygen extraction fraction (OEF, %) are shown in **A**, while CBF/$CMRO_2$, CBF/CBV and the cerebral perfusion pressure (CPP) indexed CBF/CBV are shown in **B**. The p-values for Kruskal-Wallis tests between all groups are shown with subsequent pairwise comparisons (Dunn's test) surviving correction for multiple comparisons ($p<0.0005$) between each TBI group and healthy control participants[A], and between early and intermediate time points post TBI[B] are shown in bold. Comparisons that did not meet this threshold are not shown. Cereb: cerebellum. DGM: deep grey matter. LF: lateral frontal. MF: medial frontal. Occip: occipital. Par: parietal. Temp: temporal. L: left. Right: right.

© 2020 Yoann Launey et al. *JAMA Neurology.*

	TBI		
	Early (n=17)	Intermediate (n=54)	Late (n=19)
**CBF**	**-0·39, $p<10^{-4}$**	**-0.34, $p<10^{-4}$**	**-0.51, $p<10^{-4}$**
**CBV**	0.12, p=0.05	**0.19, $p<10^{-4}$**	0.12, p=0.04
**$CMRO_2$**	**-0.53, $p<10^{-4}$**	**-0.73, $p<10^{-4}$**	**-0.78, $p<10^{-4}$**
**OEF**	0.07, p=0.14	**-0.16, p=0.001**	-0.09, p=0.05

eTable 3: Linear mixed effects model for comparison of regional physiological derangements

Using a linear mixed effects regression of log transformed data whilst adjusting for region of interest as a factor and stratifying by subject (random intercept), we compared models using data from healthy control participants and patients after traumatic brain injury (TBI) using chi square tests. Data shown are log transformed covariate effect estimates for cerebral blood flow (CBF), cerebral blood volume (CBV), cerebral oxygen metabolism ($CMRO_2$) and oxygen extraction fraction (OEF). Significant differences between patients after TBI at early, intermediate and late time points and healthy controls that survive correction for multiple comparisons are highlighted in bold.

© 2020 Yoann Launey et al. *JAMA Neurology.*

A

	CBF				***CBV***				***$CMRO_2$***				***OEF***			
	**Early**	**Inter**	**Late**	**p-value**	**Early**	**Inter**	**Late**	**p-value**	**Early**	**Inter**	**Late**	**p-value**	**Early**	**Inter**	**Late**	**p-value**
Cereb L	28 (27-32)	33 (29-37)	29 (24-32)	0.223	4.2 (3.9-4.6)	4.4 (3.9-5.1)	4.4 (3.8-4.9)	0.607	88 (73-92)	68 (60-79)	66 (58-78)	0.223	38 (37-52)	34 (30-40)	36 (33-42)	0.223
Cereb R	31 (26-35)	34 (30-41)	30 (24-33)	0.223	4.0 (3.6-4.2)	4.1 (3.5-4.3)	3.5 (3.4-4.0)	0.607	86 (76-94)	69 (64-81)	70 (57-76)	0.607	40 (38-50)	34 (29-38)	34 (30-42)	0.223
DGM L	26 (22-28)	26 (22-27)	24 (20-26)	0.368	2.5 (2.3-2.7)	2.7 (2.3-3.2)	2.7 (2.5-3.0)	0.441	71 (64-82)	55 (53-64)	51 (45-61)	0.097	44 (38-54)	36 (34-40)	35 (32-40)	0.097
DGM R	26 (23-29)	25 (22-27)	22 (19-28)	0.097	3.1 (2.9-3.4)	3.1 (2.9-3.7)	3.1 (2.7-3.4)	0.441	65 (61-79)	55 (51-58)	54 (44-56)	0.097	43 (36-47)	36 (34-40)	37 (32-39)	0.264
LF L	28 (26-30)	27 (27-27)	28 (27-28)	-	3.4 (3.2-3.6)	2.9 (2.9-2.9)	3.6 (3.5-3.7)	-	98 (94-101)	81 (81-81)	77 (70-83)	-	49 (46-52)	49 (49-49)	49 (47-51)	-
LF R	27 (23-31)	25 (24-28)	19 (17-21)	0.097	2.6 (2.4-3.0)	2.7 (2.3-3.0)	2.9 (2.6-3.0)	0.264	78 (74-92)	62 (61-67)	59 (45-71)	0.050	50 (41-61)	42 (40-45)	44 (40-47)	0.097
MF L	24 (22-27)	24 (22-27)	22 (19-26)	0.368	3.5 (2.7-3.7)	3.1 (2.7-3.6)	3.2 (3.0-3.5)	0.368	73 (71-81)	63 (57-67)	66 (58-73)	0.368	51 (44-56)	42 (39-48)	46 (42-47)	0.368
MF R	26 (23-29)	24 (22-27)	21 (16-28)	0.223	3.1 (2.9-3.4)	3.3 (3.1-3.4)	3.6 (3.0-4.3)	0.223	77 (75-92)	62 (59-67)	63 (51-65)	0.135	50 (43-57)	42 (41-43)	44 (41-46)	0.223
Occip L	25 (22-30)	26 (23-30)	26 (23-28)	0.264	4.5 (4.1-4.8)	4.3 (3.9-5.0)	4.1 (4.0-4.5)	0.717	81 (62-83)	61 (52-69)	64 (53-77)	0.717	46 (38-58)	39 (35-45)	37 (36-44)	0.368
Occip R	27 (25-31)	27 (23-29)	25 (20-27)	0.097	5.8 (5.1-6.3)	6.2 (5.7-6.5)	5.2 (5.0-5.7)	1	75 (60-82)	60 (55-67)	56 (44-74)	0.717	38 (36-51)	40 (38-43)	38 (35-44)	0.717
Par L	25 (21-28)	26 (25-27)	24 (21-26)	0.264	3.3 (3.0-3.6)	3.4 (3.2-3.8)	3.4 (3.3-3.7)	0.717	78 (76-87)	64 (58-68)	60 (49-68)	0.097	50 (42-62)	41 (36-44)	41 (37-46)	0.097
Par R	25 (22-29)	24 (22-26)	22 (19-26)	0.097	3.7 (3.2-4.1)	3.9 (3.3-4.2)	3.5 (3.0-4.0)	0.717	75 (68-84)	60 (56-66)	49 (46-64)	0.097	46 (42-57)	43 (40-45)	37 (36-43)	0.097
Temp L	30 (26-30)	25 (23-26)	24 (24-24)	0.368	3.7 (3.6-4.0)	3.5 (3.4-3.6)	4.0 (3.9-4.1)	0.368	80 (68-81)	66 (60-71)	60 (50-70)	0.368	40 (39-41)	44 (42-47)	40 (35-44)	0.368
Temp R	24 (22-28)	25 (23-27)	20 (17-22)	0.135	3.7 (3.3-4.5)	3.6 (3.3-4.4)	3.5 (3.2-3.7)	0.223	76 (71-83)	61 (58-62)	48 (44-55)	0.135	46 (41-53)	41 (37-46)	43 (39-44)	0.607

© 2020 Yoann Launey et al. *JAMA Neurology.*

B

	CBF/CMRO₂ ratio				*CBF/CBV ratio*				*CBF/CBV/CPP*			
	**Early**	**Inter**	**Late**	**p-value**	**Early**	**Inter**	**Late**	**p-value**	**Early**	**Inter**	**Late**	**p-value**
Cereb L	0.34 (0.29-0.43)	0.45 (0.40-0.56)	0.42 (0.33-0.53)	0.135	7.2 (5.7-7.8)	7.2 (6.6-8.4)	6.8 (6.4-7.3)	0.223	0.10 (0.08-0.10)	0.09 (0.07-0.12)	0.08 (0.08-0.09)	0.368
Cereb R	0.38 (0.29-0.46)	0.46 (0.40-0.58)	0.45 (0.33-0.57)	0.135	8.6 (7.0-9.7)	8.7 (8.1-9.7)	7.2 (6.7-9.4)	0.223	0.11 (0.09-0.12)	0.11 (0.08-0.13)	0.10 (0.09-0.11)	0.368
DGM L	0.31 (0.29-0.42)	0.40 (0.39-0.49)	0.49 (0.33-0.54)	0.717	10.3 (10.2-10.8)	9.0 (8.3-10.8)	8.3 (7.0-9.8)	0.097	0.14 (0.12-0.14)	0.12 (0.09-0.14)	0.09 (0.08-0.11)	0.223
DGM R	0.37 (0.32-0.47)	0.45 (0.38-0.52)	0.43 (0.39-0.55)	0.717	8.7 (7.6-8.9)	7.6 (7.0-8.5)	8.2 (6.5-9.0)	0.097	0.11 (0.11-0.12)	0.10 (0.07-0.11)	0.10 (0.08-0.12)	0.223
LF L	0.29 (0.28-0.30)	0.33 (0.33-0.33)	0.38 (0.33-0.42)	-	8.4 (8.4-8.4)	9.3 (9.3-9.3)	7.6 (7.5-7.7)	-	0.12 (0.11-0.12)	0.09 (0.09-0.09)	0.11 (0.11-0.11)	-
LF R	0.31 (0.28-0.46)	0.40 (0.34-0.43)	0.32 (0.28-0.40)	0.368	10.3 (9.8-10.7)	9.2 (8.6-10.2)	6.7 (6.2-7.4)	0.097	0.14 (0.13-0.15)	0.13 (0.10-0.14)	0.08 (0.08-0.09)	0.135
MF L	0.32 (0.28-0.41)	0.36 (0.34-0.45)	0.34 (0.30-0.41)	0.368	7.6 (6.9-8.5)	7.6 (6.8-9.4)	7.2 (6.2-8.0)	0.368	0.11 (0.09-0.11)	0.10 (0.08-0.11)	0.08 (0.07-0.08)	-
MF R	0.33 (0.28-0.37)	0.39 (0.34-0.41)	0.38 (0.30-0.46)	0.135	8.2 (7.6-8.6)	7.1 (6.4-7.4)	6.4 (5.3-6.9)	0.223	0.10 (0.10-0.12)	0.09 (0.08-0.10)	0.07 (0.07-0.08)	0.368
Occip L	0.32 (0.28-0.38)	0.40 (0.35-0.50)	0.44 (0.33-0.48)	0.368	5.4 (5.3-6.1)	6.3 (4.8-7.1)	5.9 (5.4-7.0)	0.097	0.08 (0.07-0.09)	0.07 (0.06-0.09)	0.07 (0.06-0.09)	0.223
Occip R	0.36 (0.29-0.48)	0.39 (0.35-0.47)	0.43 (0.33-0.51)	0.717	4.6 (3.9-6.2)	4.3 (3.5-4.9)	4.8 (4.1-5.7)	0.097	0.06 (0.05-0.08)	0.05 (0.04-0.07)	0.06 (0.05-0.07)	0.135
Par L	0.29 (0.25-0.33)	0.40 (0.37-0.44)	0.44 (0.31-0.50)	0.368	7.1 (6.4-8.2)	7.6 (6.6-8.1)	7.2 (5.8-7.3)	0.097	0.10 (0.08-0.10)	0.09 (0.09-0.11)	0.08 (0.07-0.09)	0.135
Par R	0.31 (0.28-0.46)	0.37 (0.34-0.43)	0.45 (0.34-0.47)	0.717	7.3 (6.7-7.6)	6.5 (5.9-7.2)	6.7 (5.3-6.7)	0.097	0.09 (0.09-0.11)	0.09 (0.07-0.09)	0.07 (0.06-0.08)	0.135
Temp L	0.37 (0.33-0.45)	0.37 (0.33-0.40)	0.44 (0.37-0.51)	0.368	7.0 (6.7-7.6)	7.3 (6.6-7.4)	6.0 (5.8-6.1)	0.368	0.10 (0.09-0.10)	0.08 (0.07-0.10)	0.07 (0.07-0.07)	0.368
Temp R	0.30 (0.28-0.40)	0.44 (0.35-0.45)	0.36 (0.34-0.42)	0.607	6.9 (6.3-7.9)	6.1 (6.0-7.1)	5.3 (5.2-5.8)	0.223	0.09 (0.08-0.11)	0.09 (0.08-0.10)	0.06 (0.06-0.07)	0.135

eTable 4: Temporal pattern of regional physiological derangements from serial data within patients

Data shown are median (interquartile range) for 17 patients after traumatic brain injury who underwent serial imaging on two or more occasions within 24 hours (Early), between day 2 – 5 (Inter) and day 6 – 10 (Late) post injury from 14 regions of interest (ROI) covering the whole brain excluding the brain stem. Regional cerebral blood flow (CBF, ml/100ml/min), cerebral blood volume (CBV, ml/100ml), cerebral oxygen metabolism ($CMRO_2$, µmol/100ml/min) and oxygen extraction fraction (OEF, %) are shown in **A**, while CBF/$CMRO_2$, CBF/CBV and the cerebral perfusion pressure (CPP) indexed CBF/CBV are shown in **B**. The p-values for the Friedman's test between serial data obtained within the same patient at different time points are shown. Cereb: cerebellum. DGM: deep grey matter. LF: lateral frontal. MF: medial frontal. Occip: occipital. Par: parietal. Temp: temporal. L: left. Right: right.

© 2020 Yoann Launey et al. *JAMA Neurology.*

Correlation	HC n=20	TBI Total (n=90)	p-value[A]	TBI Early (n=17)	TBI Intermediate (n=54)	TBI Late (n=19)	p-value[B]
$CBF/CMRO_2$	0·75 (0·67–0·86)	0·44 (0·25–0·64)	$<10^{-4}$	**0·56[C]** (0·19–0·72)	**0·39[D]** (0·23–0·55)	0·60 (0·29–0·82)	$<10^{-4}$
CBF/CBV	0·36 (0·08–0·56)	0·35 (0·11–0·61)	0·70	0·13 (0·04–0·51)	0·34 (0·12–0·62)	0·43 (0·15–0·60)	0·53

eTable 5: Flow metabolism coupling and the association between cerebral blood flow and volume

The correlation coefficients between cerebral blood flow and cerebral oxygen metabolism ($CBF/CMRO_2$) and CBF and cerebral blood volume (CBF/CBV) were calculated using the Spearman rank correlation test and are expressed as *Rho* (interquartile range) for healthy control participants (HC) and patients after traumatic brain injury (TBI). [A]Mann-Whitney test between all patients after TBI and healthy controls. [B]Kruskal-Wallis test between all groups, with subsequent post hoc Dunn's tests surviving correction for multiple comparisons within the group are shown in bold. [C]p<0·01; [D]$p<10^{-4}$ for comparison with healthy controls.

© 2020 Yoann Launey et al. *JAMA Neurology.*
